# Robotic-assisted beating heart surgery: a comprehensive review

**DOI:** 10.3389/fmed.2026.1916578

**Published:** 2026-09-29

**Authors:** Omar Abuzied, Momen Abayazid

**Affiliations:** 1 Control and Instrumentation Engineering Department, King Fahd University of Petroleum and Minerals, Dhahran, Saudi Arabia; 2 Interdisciplinary Research Center for Bio-Systems and Machines, King Fahd University of Petroleum and Minerals, Dhahran, Saudi Arabia

**Keywords:** beating heart surgery, cardiac motion compensation, clinical translation, haptic teleoperation, motion prediction, robotic minimally invasive surgery, shared control, visual servoing

## Abstract

Robotic-assisted minimally invasive cardiac surgery offers the potential to perform off-pump beating heart procedures without cardiopulmonary bypass, but active closed-loop algorithmic motion compensation, as distinct from image-stabilization, motion-tracking, and mechanical-stabilization approaches that have seen limited clinical use, has not yet entered human clinical trials despite two decades of research. This scoping review, conducted using the PRISMA-ScR framework, addresses three questions: what are the quantitative performance limits of current sensing, prediction, and control methods; what is the state of clinical translation; and what barriers prevent first-in-human deployment. Studies were included if they reported methods or quantitative results for sensing, predicting, or compensating cardiac motion in robotic surgery (61 studies included); non-robotic stabilization studies and non-English publications were excluded. Four databases were searched from 1998 to May 2026; formal risk of bias assessment was not performed, consistent with PRISMA-ScR guidance. Results were synthesized narratively by thematic area. Key results include: dual Fourier-series EKF achieves 0.73–0.81 mm RMSE at 180 ms horizon; neural network approaches reduce this by 60% at 160 ms latency; cascade MPC-AOB maintains <0.08 N RMS force error across threefold stiffness variation; and shared canonical control reduces surgeon tracking error by 41%. The primary barrier is a systems integration deficit rather than any isolated algorithmic shortcoming. Six open challenges are classified by difficulty. The evidence base is limited by predominant porcine model validation. This review was not pre-registered, and received no external funding.

## 1 Introduction

### 1.1 Overview

Robotic-assisted minimally invasive surgery (MIS) has transformed cardiac procedures, improving dexterity and precision while reducing trauma, and is increasingly used for mitral valve repair, coronary artery bypass grafting (CABG), and selected congenital operations (1). The principal obstacle to beating-heart intervention is the intrinsic motion of the heart, which is continuous, large-amplitude, non-rigid, nonlinear, patient-specific, and at times arrhythmic. This motion challenges precise manipulation and motivates methods that, integrated with robotic instruments, let the surgeon operate on an effectively stabilized surface (2), achieving the tracking precision required for procedures such as CABG (3).

Over two decades, substantial effort has been devoted to estimating, predicting, and compensating cardiac motion. Vision-based tracking (4, 5), ultrasound-guided compensation (6, 7), Kalman filtering and predictive control (3), and adaptive model-reference control (8) have shown promise in laboratory and limited in-vivo settings.

Yet clinical integration of autonomous or semi-autonomous compensation remains limited; robotic cardiac surgery still relies on telemanipulation rather than real-time motion cancellation (1). Conventional procedures achieve sub-millimeter stability by arresting the heart with cardioplegia and cardiopulmonary bypass (CPB), but CPB carries inflammatory, neurological, and procedural costs. Off-pump approaches avoid CPB at the price of reintroducing nonlinear cardiac motion, so motion compensation promises both improved precision and broader, lower-burden access to beating-heart procedures.

Existing solutions face four recurring limitations: many algorithms assume quasi-periodic motion and degrade under arrhythmia (3); vision is vulnerable to occlusion, reflection, and blood contamination (4); prediction-based control is sensitive to latency and phase mismatch; and most validation is performed under controlled laboratory conditions that neglect respiratory coupling, micro-movements, and long-duration variability. Real-time tracking of fast, deformable targets under surgical constraints thus remains an open systems-level challenge (9).

This review evaluates the clinical and technical progress of cardiac motion compensation in robotic MIS, analyzing sensing, motion modeling, control architectures, validation, and surgeon-centered considerations, and examining the gap between experimental demonstration and clinical practice. It addresses three questions: 1. What sensing, prediction, and control methods have been proposed, and what are their quantitative limits? 2. What is the state of clinical translation, and what separates pre-clinical validation from first-in-human deployment? 3. What engineering, research, and institutional barriers must be closed?

Previous reviews are partial. Yang and Kobayashi (10) surveyed vision-based tracking for surgical navigation but not control; Mohd Fua’ad et al. (11) surveyed 23 machine-learning cardio-respiratory prediction studies but excluded sensing, control, and translation; and Poddi and Rungatscher (12) reviewed the clinical state of minimally invasive cardiac surgery without the control algorithms needed for compensation. No prior review spans the full chain of motion characterization, sensing, prediction, control, and safety, together with an explanation of the lack of clinical translation. The main contributions of the paper are fourfold. Specifically, the paper makes an effort to provide the first review of the field that encompasses all of the stages of the compensation chain: motion characterization, sensing, prediction, control, and safety within a single systems-level framework; it provides a comparison of sensing, prediction, and control methods (Tables 1–3); it identifies the deficit in integrating the components of the system as the primary challenge for translation into clinical treatments, as opposed to any single component of the system as well as classifies the six open challenges by their underlying nature (engineering, research, or institutional); and, finally, it offers four recommendations regarding how to proceed with first-in-human trials of these approaches.

**Table 1:** Comparison of sensing modalities for cardiac motion estimation in robotic-assisted surgery.

Modality	Spatial resolution	Temporal rate/latency	Contact required?	Occlusion robustness	Procedure suitability	Key limitation
Stereo endoscope (vision)	∼1–2 mm (3D)	50–83 Hz; ∼12 ms	No	Low (instrument, blood, smoke)	CABG, TECAB (epicardial)	Specular reflections; textureless zones
3D Ultrasound	∼1–2 mm	24–28 Hz; 50–100 ms delay	No (intracardiac probe)	High (penetrates tissue)	Intracardiac (mitral valve, catheter)	Low frame rate; processing delay
ECG/airflow signal	Temporal only	>500 Hz; ∼20–25 ms (custom amp)	No (surface electrodes)	N/A (no spatial info)	Any (as auxiliary input)	No spatial information; arrhythmia degrades prediction
Force sensor	Force only	>1 kHz; minimal	Yes (requires contact)	N/A (contact dependent)	Contact tasks (suturing, needle insertion)	Mixed signal; contact required
Fiducial markers (optical)	2–3 mm (3D)	30–50 ms per frame	No (marker on instrument)	Moderate (marker must be visible)	Open-heart, instrumented tools	Sterility; adds weight; instrument tracking only
3D Echo (respiratory)	∼3–4 mm	∼Real-time	No (transthoracic probe)	High (respiratory component)	Image-guided catheterization	Respiratory motion only; not cardiac-cycle resolved

**Table 2:** Comparison of cardiac motion prediction algorithms.

Method	Prediction horizon	RMSE	Arrhythmia robustness	Real-time?	Validation
AR/fading AR (2, 47)	1-step ahead	Does not achieve sub-$\sigma_{R}$ error; degrades rapidly for ΔHR>4 bpm	None; model breaks under irregular rhythm	Yes; low computational cost	In-vitro (1-DOF MCI, mitral valve annulus simulation) (2)
WFLC (2, 47)	1-step ahead	Accuracy comparable to EKF at small ΔHR; slow convergence above 4 bpm	None	Yes	In-vitro (mitral valve annulus data, comparative study (2))
Dual-component Fourier-EKF (5, 18)	0.18 s and 3 s	0.8076 mm RMSE at 0.18 s (single dataset); 0.73–0.81 mm across 3 datasets at 0.18 s; 0.80–1.02 mm at 3 s	Transient spike (∼0.17 s) then rapid re-convergence after isolated beat; struggles with sustained arrhythmia	Yes; 83–125 Hz stereo endoscope	In-vivo porcine (DAVINCI, LIRMM1, LIRMM2 datasets)
DKF with IOSF (23, 37)	50 frames (∼2 s)	0.584 mm RMSE vs. 0.681 mm for joint EKF on in-vivo da Vinci data	More robust than EKF; no “self-coincident” divergence under frequency step changes	Yes; da Vinci stereo endoscope	In-vivo (da Vinci CABG sequences); simulated frequency step changes (23)
Adaptive LS (generalized predictor) (20)	25 ms (4-point horizon)	0.162–0.404 mm RMSE across 3 calves, worst-case experimental (generalized estimator)	Stable across pacemaker-driven 95–152 bpm; degrades at abrupt step changes	Yes; 2 kHz control loop (133 Hz optimal downsampled)	In-vivo calf (sonomicrometry, 3-DOF robotic testbed)
Master-slave UKF/ANM (3)	Online (not specified)	20.1 and 22.4μm 3D RMSE (calf datasets) vs. 227.4μm (AR), 131.5μm (FS-EKF); 18.1 and 15.7μV RMSE on MIT-BIH PVC and AF/flutter datasets	Explicitly designed for arrhythmia; Slave UKF adaptively inflates process noise on anomalous beats; validated on clinical PVC (106m) and AF/flutter (203m) ECG recordings	Yes (online adaptive)	In-vivo calf (sonomicrometry); MIT-BIH arrhythmia database (ECG signals)
NAR recurrent neural network (50)	160 ms (US pipeline delay)	∼60% lower MAE and RMSE vs. EKF (fixed HR: MAE 0.376 vs. 0.959 mm); ∼70% lower (varying HR)	Not evaluated; requires retraining for new conditions	Offline training required; online inference at 25 Hz US frame rate	In-vitro (US simulator, 1-DOF cam + voice coil actuator, plastisol tissue, water tank)
PMRAC + neural network (8)	Online (rolling 20-sample window retraining)	<0.15 mm RMSE on real human heart dataset	Not evaluated	Yes; continuous retraining on rolling window	Real human heart dataset (vision-based, Lucas-Kanade tracking)

**Table 3:** Validated systems for robotic and computer-assisted beating heart motion compensation.

System/study	Target procedure	Guidance modality	Validation level	Key result	Year
Nakamura et al.—Heartbeat Synchronization	MIDCAB epicardial surface	High-speed camera (955 fps) + AR tracking	Laboratory (artificial oscillating laser point)	First robotic heartbeat synchronization; residual ∼0.5 mm deviation	2001
Gründeman et al.—EndoStarfish/endoOctopus	Multivessel TECAB posterior/inferior CABG	Da Vinci + endoscopic CO monitoring	In-vivo (porcine)	Coronary target restrained to ∼1×1 mm; 16 anastomoses completed	2003
Lemma et al.—Mechanical Stabilizer Benchmark	Off-pump CABG LAD/PDA/OM	Stereophotogrammetry + fiducial markers	In-vivo (porcine)	Residual motion 0.8–1.1 mm (SD); all three stabilizers similar performance	2005
Yuen et al.—3DUS Mitral Valve MCI	Beating heart mitral annuloplasty	3D ultrasound (28 Hz) + EKF prediction	In-vitro	1.8 mm RMS tracking error; anchor failure rate ∼60% lower vs. solid instrument	2008/2009
Kesner & Howe—Motion Compensation Catheter	Mitral valve intracardiac repair via catheter	3D ultrasound + PID (1 kHz)	In-vivo (porcine)	<1 mm RMSE tracking; first robotic catheter for intracardiac motion compensation	2011
Yuen et al. (2013)—Robotic Tissue Tracking	Beating heart mitral valve surgery	3DUS flashlight tracker + EKF	In-vivo (porcine)	0.8–1.0 mm RMSE system vs. 3.2 mm manual (70% less error); 68 ms 3DUS delay compensated by EKF	2013
Stenmark et al.—Live Open-Heart Tracking	Pediatric ASD/PAPVC open surgery	ArUco marker vision tracking (laptop)	5 live pediatric open-heart procedures	2–3 mm 3D accuracy; 25 fps on standard laptop; negligible inconvenience	2022
Balkhy—Robotic Beating-Heart TECAB Program	Single/multivessel totally endoscopic CABG	Da Vinci Endowrist Stabilizer + C-Port	Clinical (>550 cases, 7-year program)	75%+ patients opioid-free by week 1; return to work mean 14 days; 10-yr patency documented	2021
Baudo & Torregrossa—RA-CABG Review	Robotic MIDCAB + hybrid TECAB	Da Vinci (multiple platforms)	Clinical (11,135 MICS-CABG cases)	95.2% 1-yr survival; 93.4% freedom from MACE; 4.7% conversion rate	2025
Wah—robotic Revolution Review	Mitral valve, CABG, AVR, transplant	Da Vinci Xi + emerging platforms	Clinical (multi-institutional review)	High-volume centers: better outcomes; learning curve surmountable at 10–50 cases	2025

### 1.2 Review scope and methodology

This review follows the PRISMA-ScR (Preferred Reporting Items for Systematic Reviews and Meta-Analyses, extension for Scoping Reviews) framework (13), surveying the sensing, prediction, and control methods proposed for beating-heart movement during minimally invasive procedures rather than performing a quantitative meta-analysis of treatment outcomes. This review was not pre-registered and a protocol was not prepared in advance.

A search of four databases (IEEE Xplore, PubMed, Scopus, and Google Scholar) was performed between November 2025 and May 2026 (all last searched in May 2026) for literature published between 1998 and 2026. The Boolean search string was: (*“beating heart surgery”* OR *“cardiac motion compensation”* OR *“heart motion prediction”*) AND (*“robotic surgery”* OR *“robot-assisted”* OR *“teleoperation”* OR *“visual servoing”* OR *“force control”*), with additional terms *“Kalman filter cardiac”* and *“motion synchronization surgery”*. No filters were applied within the database interfaces; the publication range and English-language restriction were enforced during screening. Reference lists of key articles were also searched for seminal work (1, 10–12, 14–16).

Articles were included if they addressed sensing, predicting, or compensating cardiac or cardio-respiratory motion during robotic surgery, or reported relevant quantitative or theoretical contributions; studies limited to manual or non-robotic stabilization devices, or not in English, were excluded. Included studies were grouped thematically into sensing (Section 3), prediction (Section 4), control (Section 5), and clinical translation (Section 6); the screening and selection process is summarized in Figure 1. Title/abstract screening and full-text assessment were performed by a single reviewer (O.A.) without automation tools, with data extracted from full-text articles (or, where full text was unavailable, from abstracts). Primary outcomes were prediction RMSE and horizon, control tracking error and force-regulation accuracy, and validation level (laboratory, pre-clinical, or clinical); secondary variables included sensing modality, robotic platform, year, and study design.

**Figure 1:**
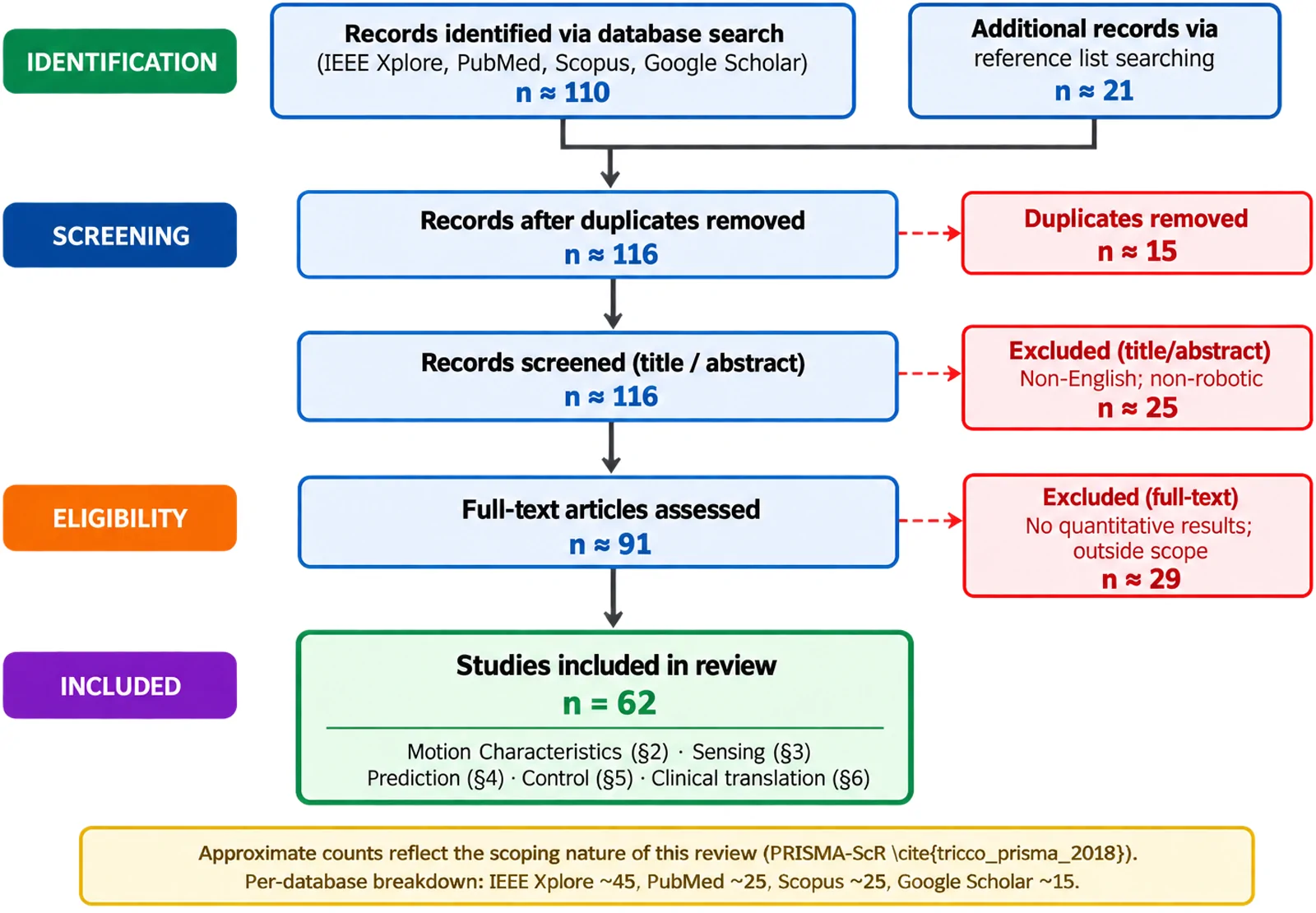
PRISMA-ScR flow diagram for the scoping review; Search period: November 2025–May 2026 ⋅ Publication range: 1998–2026; Databases: IEEE Xplore, PubMed, Scopus, Google Scholar.

Consistent with PRISMA-ScR guidance for scoping reviews (13), risk-of-bias, effect-size, statistical-synthesis, and certainty-of-evidence assessments were not performed; the review synthesizes methodological approaches and performance metrics rather than pooling clinical outcome data. Information regarding individual excluded studies is not required for scoping reviews; the criteria for the studies to be included in the review are stated above.

### 1.3 Paper organization

The paper is organized as follows. Section 2 characterizes the multi-source dynamics of cardiac surface motion and the physical requirements any compensation system must satisfy; Section 3 surveys the available sensing modalities; Section 4 reviews five paradigms of motion prediction; Section 5 examines the four principal control architectures; Section 6 assesses clinical translation, identifies six open challenges, and outlines prioritized future directions; and Section 7 synthesizes the cross-cutting, system-level lessons and conclusions.

## 2 Cardiac motion: characteristics and sources of disturbance

The safety and feasibility of robotic-assisted beating heart surgery hinge on understanding cardiac motion and the surgical environment. Unlike open surgery under CPB, off-pump procedures expose the surgeon and the robot to a continuously moving myocardial surface whose dynamics span multiple sources and time scales. The aggregate motion at a point of interest (POI) on the epicardial surface is therefore not a simple sinusoid but a superposition of quasi-periodic components driven by cardiac contraction and respiratory excursion, plus secondary disturbances such as anaesthetic-modulated physiological variability and tissue compliance under instrument contact.

### 2.1 Cardiac contraction dynamics

#### 2.1.1 Amplitude and velocity

Epicardial motion has been measured in open-chest porcine models and via stereo-endoscope during CABG. Riviere et al. (17) tracked natural markers on a porcine heart and reported peak velocities exceeding 0.1m/s and accelerations of $10\;m/s^{2}$ under normal sinus rhythm at 60 bpm, roughly doubling (0.2 m/s, $20\;m/s^{2}$) after adrenaline injection. Richa (18) reported, for the LIRMM2 porcine dataset (125 Hz, 15 s, unstabilized), displacement ranges of [−2.9,4.6], [−1.8,1.3], and [37.5,49.8] mm in x, y, and z, the dominant z-axis reflecting apex-to-base contraction. These are broadly consistent with (19), who characterized mitral-annulus motion in sheep as a travel of roughly 20 mm with centroid velocity and acceleration maxima of 210 mm/s and $3,800\;\mathrm{mm}/s^{2}$ at 76 bpm. Active relative motion canceling studies by (20) found peak-to-peak amplitudes of 8.39 mm (RMS displacement 3.55 mm) at constant rate and 7.43 mm (3.38 mm) under paced varying rate, indicating that amplitude is largely preserved across physiologically relevant rates even as the spectral centroid shifts.

#### 2.1.2 Spectral characterization

Measured cardiac motion has a multi-harmonic structure that motivates Fourier-series models; the fundamental cardiac frequency under normal sinus rhythm is 1–2 Hz. Nakamura et al. (21) [cited in (2, 19)] identified dominant components at 1.5 and 3.0 Hz in porcine epicardium, and mitral-annulus decomposition confirmed components at 1.3, 2.6, and 5.2 Hz with decreasing amplitude at higher frequencies.

#### 2.1.3 Quasi-periodicity and non-stationarity

A defining property of cardiac motion is its quasi-periodicity: the waveform is approximately, but not exactly, periodic, with slow variation in amplitude, phase, and instantaneous frequency due to autonomic activity, respiratory coupling, and beat-to-beat heart-rate variability. Recognized early (5, 22, 23), this property is central to Fourier-series prediction models, where the coefficients must be treated as slowly time-varying rather than fixed, requiring adaptive estimation. Non-stationarity can also manifest as abrupt frequency shifts; in (2), the extended Kalman filter (EKF) predictor remained in phase with the target under beat-to-beat variation of $\sigma_{HR}=1.0$ to 4.0 bpm drawn from MIT-BIH Normal Sinus Rhythm records.

#### 2.1.4 Mathematical representation of cardiac motion

To capture quasi-periodicity, Fourier series with time-varying coefficients are used. A non-stationary Fourier series was used by (22) to obtain full 3D POI motion; later work separated the cardiac and respiratory contributions into a dual-component Fourier series (Equation 2.1) (5, 18):

$$\begin{array}{rl} p(t,f)= & \Sigma_{h=1}^{H_{r}}[a_{h}\sin(h\sigma \omega_{r})+b_{h}\cos(h\Sigma \omega_{r})]+c_{r}\\ & +\Sigma_{h=1}^{H_{c}}[d_{h}\sin(h\Sigma \omega_{c})+e_{h}\cos(h\Sigma \omega_{c})], \end{array}$$ (2.1)

where $H_{r}$ and $H_{c}$ are the numbers of respiratory and cardiac harmonics, $\omega_{r}$ and $\omega_{c}$ the instantaneous fundamental frequencies, and $f=[a_{1},\ldots ,aH_{r},b_{1},\ldots ,bH_{r},c_{r},d_{1},\ldots ,dH_{c},e_{1},\ldots ,eH_{c}{]}^{T}$ the coefficient vector. This formulation, used by Richa (5) and Yang et al. (23), is the most widely used parameterization in robotic surgery. An alternative used by Yuen et al. (2) is a dual time-varying Fourier series (DTVFS) expressed through harmonic amplitudes and phases in Equation 2.2:

$$y(t)=c+\Sigma_{i=1}^{m}r_{i}(t)\sin\;(i\omega (t)t+\Phi_{i}(t))$$ (2.2)

This form is equivalent to Equation 2.1 when its time-varying components are split into real and imaginary parts, but its parameters are physically interpretable and directly measurable from the patient’s ECG and Doppler signals.

### 2.2 Respiratory motion coupling

The second major source of epicardial displacement is respiratory motion, created by the diaphragm and rib cage, which increase intrathoracic volume and produce cranio-caudal translation and rotation of the heart within the pericardial sac. McClelland et al. (24) report that the diaphragm and rib cage contribute differently depending on posture, breathing depth, and individual anatomy.

#### 2.2.1 Respiratory displacement magnitude

Respiratory displacement exceeds contractile displacement in the cranio-caudal direction. King et al. (25) report up to 25 mm of breathing-induced movement, referencing the MRI studies of McLeish et al. (26); the effect is stronger under mechanical ventilation with deeper tidal volumes. Spectral analysis (17) places this slow respiratory movement at approximately 0.25 Hz, relative to roughly 1 Hz for cardiac movement.

#### 2.2.2 Frequency characteristics and spectral separation

For patients undergoing mechanical ventilation, the frequency at which the respiratory movements occur is controlled by the mechanical ventilator. For example, mechanical ventilators can be set to provide respiratory rates of 0.2 to 0.4 Hz (18). Moreover, motion analyses of the heart indicate that the dominant frequencies in all axes are between 0 and 5 Hz in all of the experimental datasets. More specifically, the fundamental frequency within the heart at the lowest range of frequencies can be seen as the respiratory rate (slow movement) of the heart, with the following, more prominent peak (1st harmonic), being the frequency of the heart beats. For instance, Tuna et al. (20) indicate that the first dominant frequency in the Fast Fourier Transform (FFT) of the heartbeat is 0.17 Hz for breathing movements, with the secont harmonic at 0.33 Hz, while the remaining movements in the higher frequencies are those of the cardiac motion of the heart itself.

These two components cannot be fully decoupled using linear filtering methods alone due to their nonlinear coupling (18, 27), justifying the need to model cardiac motion as a combination of both components.

#### 2.2.3 Respiratory cardiac coupling

The coupling between respiratory and cardiac motion is even more complex than discussed above. For instance, Cuvillon et al. (27), as well as Bachta et al. (28) both cited in (18) reported that the amplitude of the heartbeats of the patients is modulated according to the position of the heart within its respiratory cycle. For example, when the heart cavity within the thoracic cage is larger, there is a decrease in the amplitude of the heartbeats; the same is true for the opposite condition (when the volume within the thoracic cage is smaller). Consequently, the coefficients for the harmonic components of the heartbeats (represented in Equation 2.1 as $d_{h}$ and $e_{h}$) exhibit a slow variation with the respiratory frequency of the patient.

In addition, Bachta et al. (28) modeled the relationship between cardiac and respiratory motion by incorporating a third series of Fourier series components into their model, in addition to the existing components for the cardiac and respiratory motion Bachta et al., 2009, cited in Richa (18). Through modeling their heart rates and respiratory rates simultaneously, Bachta et al. discovered that their three-series model for heart motion beat exhibited less error than both the single-series model for cardiac motion alone, or a dual-series model in which the components for cardiac and respiratory motion were independent of one another.

Furthermore, the coupling between cardiac and respiratory motion was experimentally confirmed by authors such as Riviere et al. (17). For instance, the authors state that ”it is mainly the amplitude of the beating that is altered by the volume of the lungs”. McClelland et al. (24) reviewed respiratory surrogate models ranging from simple affine fits to patient-specific registration models, with affine fits achieving 3.3±1.1 accuracy at the cardiac level (25). The motion source contributions and their composite effect are illustrated in Figure 2.

**Figure 2:**
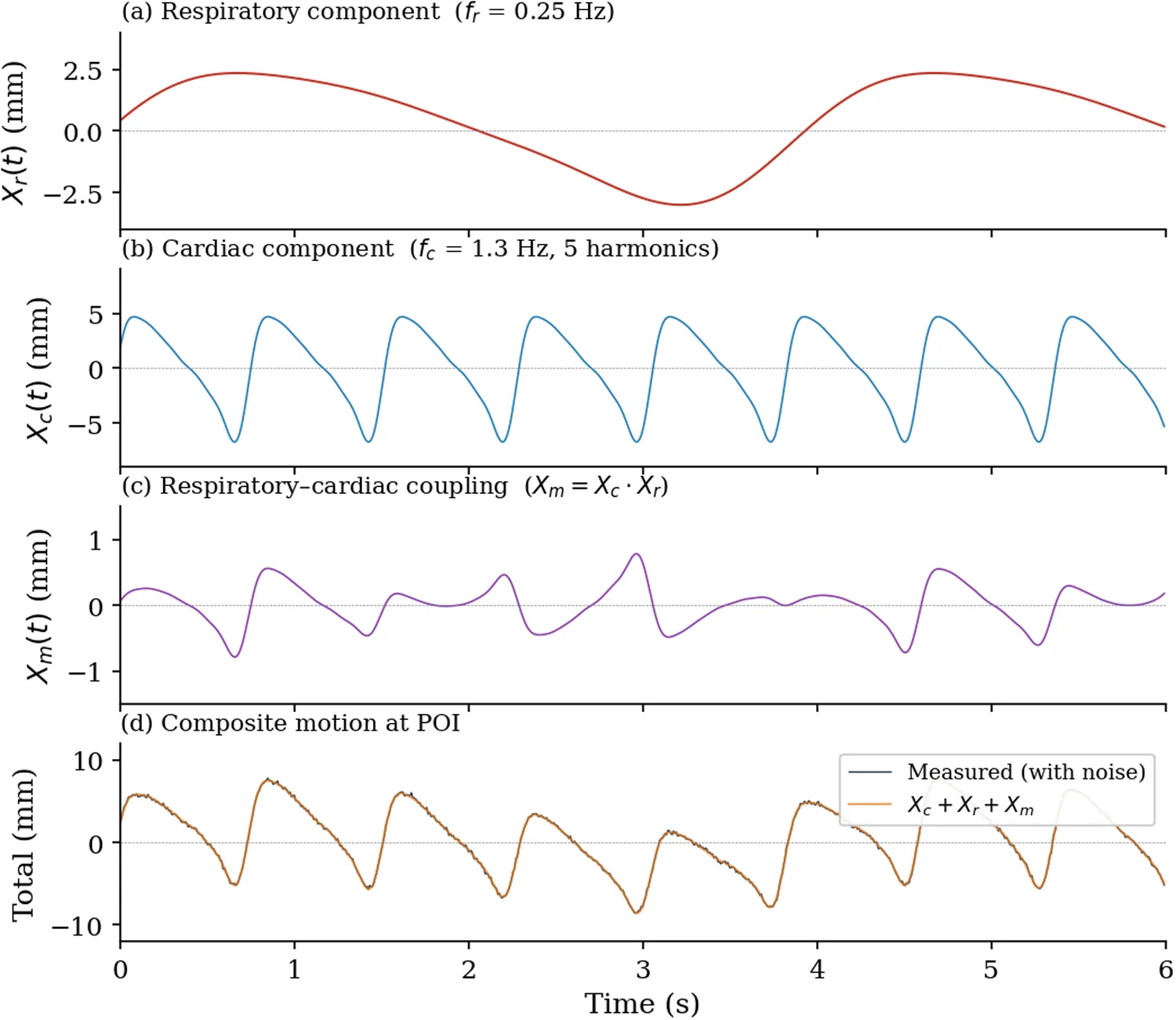
Motion source decomposition diagram.

#### 2.2.4 Implications for beating heart surgery

The repeatability of ventilated respiratory motion is often exploited; Ginhoux et al. [(29), cited in (18)] assumed the respiratory component perfectly periodic and filtered it out before modeling the cardiac component as a five-harmonic Fourier series, which is valid only under mechanical ventilation. If respiration is uncontrolled or unknown, the dual-component model of Equation 2.2 becomes necessary. Moreover, repositioning the heart with devices such as the Endo-Starfish positioner (30) alters the respiratory component, invalidating models built on pre-repositioning observations.

### 2.3 Additional motion sources

Beyond cardiac and respiratory motion, smaller disturbances can still degrade compensation by introducing motion outside the filter bandwidth or non-stationary content into the motion model.

#### 2.3.1 Residual motion from mechanical stabilization

One of the primary methods for reducing the motion of the heart is through the use of mechanical stabilizers. Devices like the Octopus (Medtronic) utilize suction forces to apply pressure to the heart, while other devices utilize pressure-based forces to stabilize the region of the heart around the coronary artery to which the device is attached. In each of these cases, however, there is still some motion that remains within the stabilized region of the heart. Richa (18) cited Lemma et al. (31) reporting measurements of 1–1.5 mm of residual motion. Such motion was attributed to both the flexibility of MIS tools that are often used in cardiac procedures, as well as the distance between the point of stabilization of the heart by the stabilizer to the site of the coronary artery intervention. Dominici et al. (32) also reported that the high pressures that are often required to stabilize the heart with mechanical stabilizers may affect hemodynamic stability, whereas suction-based stabilizers introduce issues related to tissue damage from strong suction forces.

The only documented stabilization device that utilizes robotics is the Cardiolock device (33), cited in (18). Cardiolock utilizes a piezoelectric actuator within its stabilizer to provide counteracting forces to the motion of the heart that is measured with sensors within the stabilizer; by applying these counterforces, the residual motion is reduced to levels of less than 0.3 mm. Thus, rather than moving the robotic tool according to the motion of the heart, the stabilizer itself is made active to counteract the heart’s motion. This approach, however, only compensates for the component of the motion of the heart that is perpendicular to the heart’s stabilizer shaft, as well as within the mechanical range of the piezoelectric actuator.

Finally, another issue related to the use of mechanical stabilizers is that they are only applied to the anterior (or “front” portion) of the heart. As a result, other portions of the heart must be repositioned with devices like the Endo-Starfish, which utilizes suction forces to grasp the apex of the heart and reposition the heart anteriorly (away from the body) by up to 90 degrees (30).

#### 2.3.2 Instrument–tissue interaction forces

Contact tasks such as suturing, needle insertion, and anastomosis introduce an additional apparent motion component from the interaction between the instrument tip and the myocardial tissue, independent of the free-surface motion model. Moreira et al. (34) and Liu et al. (35) modeled the epicardial tissue as a viscoelastic medium with both elastic and damping behavior, comparing linear (Kelvin-Voigt, Kelvin-Boltzmann) and nonlinear (Hunt-Crossley) models. The nonlinear Hunt-Crossley model and its advantages for force control are developed in Section 5.3.1, where it forms the basis of the force-controlled architectures.

#### 2.3.3 Beat-to-beat variability and arrhythmia

Beyond normal sinus rhythm, arrhythmia produces inherently unpredictable trajectories. Yuen et al. (6) note that the EKF-based quasi-periodic model does not extend to arrhythmia, but the ECG can signal arrhythmia onset roughly 90 ms in advance, allowing the robot to enter a failsafe hold. Liang et al. (3) instead propose a Master-Slave Kalman Filter (Section 4) whose slave filter adaptively inflates process-noise covariance on deviation from the expected trajectory, demonstrating arrhythmia robustness on two patient datasets. Pacing is an alternative mitigation (36); Tuna et al. (20) used an artificial pacemaker to hold heart rate within 95–152 bpm.

#### 2.3.4 Surgical field disturbances

The surgical environment itself is disruptive: irrigation fluid, electrocautery smoke, pooled blood, and instrument occlusion can all obstruct vision-based tracking. Specular reflections from the wet epicardium degrade landmark visibility and require illumination compensation before feature extraction (18, 35), and instrument occlusion must be bridged by temporal prediction.

## 3 Sensing modalities for heart motion estimation

### 3.1 Overview and sensing requirements

Accurate, timely measurement of the cardiac surface trajectory is the foundation of any compensation system: the sensing modality fixes spatial and temporal resolution, loop latency, workspace invasion, occlusion susceptibility, and procedure applicability. Four main modalities have been explored: endoscopic vision, three-dimensional ultrasound (3DUS), physiological-signal sensing (ECG and respiratory airflow), and contact force, with fiducial optical tracking used mainly as a ground truth; the most capable systems fuse two or more. Any modality must satisfy three conditions: a measurement rate adequate for the highest motion frequency [at least 25–30 Hz to capture 5 Hz content, though higher is preferred (18)]; latency low enough that the residual delay after predictive compensation stays within the sub-millimeter CABG error budget; and a size compatible with the crowded MIS workspace.

### 3.2 Endoscopic vision-based sensing

The endoscopic camera present in every MIS platform, including da Vinci, is the most natural and least invasive way to sense the epicardial surface, and vision has dominated compensation for CABG and totally endoscopic coronary artery bypass (TECAB). Yang and Etsuko (10) review vision-based tracking for surgical navigation.

#### 3.2.1 Feature-based tracking

The earliest methods used feature-based tracking. Ortmaier et al. (36) tracked naturally occurring landmarks (e.g., vessel bifurcations) with an affine block-matching model and a confidence measure to reject estimates during occlusion. Subsequent work explored richer descriptors such as Maximally Stable Extremal Regions (MSER), machine-learned descriptors, and anisotropic detectors (37), but feature methods depend on natural features, which are sparse where the epicardium is textureless and corrupted by specular highlights.

#### 3.2.2 Region-based and TPS deformable surface tracking

Region-based (or direct) tracking methods use the entire intensity information of a template region to estimate surface motion, circumventing the need for discrete feature correspondences. The critical design choice is the deformation model used to parametrize the surface warp. Early models that were used for this purpose included affine and projective models, B-spline transformations models [cited in (18)], and piecewise bilinear models [cited in (37)]. Each of these models are rigid models and, therefore, are not well-suited to describing the non-rigid deformations of the myocardium during contraction; they are also not robust to specular highlights and textureless areas of the epicardial surface.

The Thin-Plate Spline (TPS) model uses radial basis functions with a basis $B(r)=r^{2}\log\;(r^{2})$, and emerged as the preferred representation for cardiac surface deformation in the work of Richa, Poignet, and Bó (18, 22, 38). The TPS model is used to interpolate the three-dimensional position of any point in a region of interest on the epicardial surface from a set of control points. The three-dimensional positions of these control points are directly estimated from the stereo image pair captured by the endoscopic cameras with no disparity search performed. Control points can be placed arbitrarily on the epicardial surface, which allows the model to account for featureless areas of the epicardium by using interpolation from nearby regions with visual features. An illumination compensation technique, similar to the approach of Silveira and Malis (39), is used to compensate for the specular highlights on the epicardium before the intensity-alignment objective is minimized. This approach to tracking is able to achieve sub-pixel accuracy and was validated on both porcine and human heart sequences performed in vivo, including one TECAB procedure performed through the daVinci robotic MIS platform. For 3D tracking in the stereo scenario, three TPS functions fx, fy, fz sharing the same control point set are stacked and back-projected through the calibrated camera matrices to recover the full 3D surface position (18, 37).

#### 3.2.3 Stereo endoscopy, 3D reconstruction, and computational acceleration

The arrival of the stereo endoscope provided the basis for 3D motion estimation. Richa (18) describes the calibration of the stereo endoscope system in experiments with two high-speed DALSA cameras attached to Storz endoscopes. The baseline between the two endoscopic cameras is small, and the calibration is performed with MATLAB’s toolbox to obtain a reprojection error of less than 0.15 pixels. The system achieves frame rates of around 83.3 Hz. For clinical applications, the daVinci stereo endoscope is used at 50 Hz. The parameterization of the TPS model described by (37) allows the 3D position of each point on the endoscopic surface to be recovered by projecting back the 2D image observations of the point using the calibrated endoscopic cameras.

The 3D tracking objective for the stereo pair simultaneously minimizes the alignment error across both camera images (5, 18), and the problem is solved via the Efficient Second-order Minimization (ESM) algorithm, which offers faster convergence and a larger basin of attraction than Gauss-Newton or Levenberg-Marquardt methods by simultaneously utilizing the Jacobians of both the template and the current image.

Richa, Bo and Poignet in (38) also proposed a novel algorithm that fuses the outputs of multiple motion trackers to improve the robustness of the estimated 3D motion. For real time tracking of the endoscopic surfaces at the required high frame rates, the authors implemented their algorithm on a graphics processing unit (GPU) using NVIDIA’s CUDA framework. Their implementation achieves frame rates of up to 80 Hz (18). Finally, Stoyanov and Yang (4) used the estimated 3D positions of the endoscopic surface and rendered them into an augmented reality (AR) stabilized image that could be viewed on the endoscopic monitor by the surgeon. The beating heart on the surgical field appeared stationary during AR display while the endoscopic robot compensated for its motion.

#### 3.2.4 Occlusion handling

Occlusion from instrument shafts, blood, irrigation, and smoke is the most disruptive vision failure mode. It was addressed (38, 40) by coupling the spatial TPS tracker with the temporal dual-Fourier prediction model (Section 2): when the alignment error indicates failure, the system switches to temporal prediction and re-converges once vision returns, bridging occlusions of several seconds. The same robustness of the dual-Fourier model was demonstrated in vivo (5, 35).

### 3.3 Three-dimensional ultrasound guidance

Endoscopic vision cannot see intracardiac structures, whereas real-time 3DUS can. The Harvard group developed most 3DUS guidance work between 2007 and 2013 (2, 6, 19, 41), targeting procedures such as mitral valve annuloplasty.

#### 3.3.1 Tissue tracking in 3DUS

The mitral annulus moves approximately unidirectionally, as found by singular value decomposition of 3D annulus tracking from sheep hearts (2). Yuen et al. (41) segmented the annulus contour in volumetric ultrasound and estimated its centroid; the later “flashlight” system (6) located target tissue by detecting the instrument’s ultrasound signal and searching along the ray to the target, segmenting the tissue with an active contour.

#### 3.3.2 Noise, latency, and EKF delay compensation

In comparison to endoscopic vision, 3DUS imaging is associated with disadvantages in its frame rate and the latency of the imaging system. Yuen et al. (2) determined that the total delay of the system was between 50 and 100 ms, which was the result of the ultrasound imaging itself (about 30 ms), the data transfer between the ultrasound system to the computer network (about 30 ms), and the latency of the control system (about 10 ms). Moreover, in (6), they determined that the mitral annulus traverses over half of its movement within the first 60 ms at the onset of ventricular relaxation. Thus, naive closed-loop system will always be lagging behind the annular movement without some method of compensation.

The solution is the EKF predictor described in Section 2 (Equation 2.2). Rather than allowing the system to be controlled by the 3DUS measurements at time t, the EKF calculates a prediction of the movement of the target tissue 78 ms into the future (2, 6). The EKF also reduces the noise within the ultrasound measurements by regularizing the ultrasound measurement against a quasi-periodic model of the heart’s motion. Experimental tests in water tanks led to the reduction in position noise of the system by 30%, and the removal of the delay in the system. In in-vivo experiments, the system achieved a root mean square (RMS) measurement of 1.0 mm in the tracking of the mitral annulus. Without such a predictive filter, the error of the system was dominated by the lag of the system; manual tracking with a surgeon was estimated to lag 339 ms behind the movement of the mitral annulus, an error that was much worse than that achieved by the robotic system.

#### 3.3.3 Respiratory motion correction with 3D echocardiography

3D echocardiography can also correct respiratory motion. King et al. (25) used a transthoracic probe to determine the heart’s respiratory position and correct a pre-operative MRI roadmap during catheterization; combining roadmap–echo registration with an MRI-based affine respiratory model (24, 25) achieved 3.3±1.1 mm accuracy per chamber, vs. 4.1±2.2 mm for echo-only registration.

### 3.4 Physiological signal sensing

ECG and respiratory pressure/airflow signals are indirect but high-bandwidth, low-latency surrogates for the two principal drivers of cardiac motion. Ortmaier et al. (36) were among the first to fuse ECG, respiratory pressure, and visual landmark tracking, using a low-latency (<20 ms) differential amplifier and 500 Hz airflow sensing, with the physiological features embedded into a nonlinear predictor (17). ECG is especially valuable during rate transitions and arrhythmia, since electrical activity precedes contraction by a fixed interval and provides ∼90 ms warning of arrhythmia onset (Section 2.3.3) for a preemptive failsafe (6). Tuna et al. (20) identify ECG integration as a key improvement to adaptive filtering, yet most published systems still rely on the adaptive Fourier-EKF to track frequency and phase from motion measurements alone.

### 3.5 Force sensing

Force sensors at the end-effector indirectly sense cardiac motion through the force with which the heart distorts the contacting instrument; the cardiac-frequency component of this force can drive compensation without optical or acoustic sensors. Poignet and Cortesão (42) used a Kalman Active Observer (AOB) driven solely by force, modeling it as a second-order stochastic disturbance and estimating its active state to generate compensating commands; because it needs only force, it is immune to visual occlusion. Dominici et al. (43) validated this on a 7-DOF WAM arm, reporting a 60%–72% reduction in peak-to-peak force on the best-compensated axis. The main drawback is that force sensing requires contact, so it is unavailable during approach or withdrawal, and the force signal must be separated from the tissue’s viscoelastic effects (34, 37).

### 3.6 Fiducial marker-based tracking

Fiducial markers, placed on the heart or instruments and tracked optically, are used mainly to validate other sensors. Riviere et al. (17) used retroreflective markers and a high-speed camera to measure porcine heart motion at up to 200 bpm, and Richa (18) used epicardial markers at 125 Hz as ground truth. Stenmark et al. (44) tracked instruments during live open-heart surgery using 3D-printed ArUco “tracking dice” on DeBakey forceps imaged by an overhead camera, achieving 2–3 mm positional and $1.1\text{--}2.6^{\circ }$ orientation accuracy at 30–50 ms per frame on a standard laptop; although less accurate than the sub-millimeter requirement for instrument compensation, the system is viable in the live operating theater without specialized hardware.

### 3.7 Comparative analysis and modality selection

Table 1 summarizes the key characteristics of these modalities. Because reported metrics arise from different substrates and setups, the table maps the design space rather than ranking modalities on a common scale. Three conclusions follow. First, since no single modality satisfies minimal latency, high frame rate, deep-structure visualization, and force sensing simultaneously, sensor fusion is the logical choice for next-generation systems; Tuna et al. (20) likewise identify fusion (including sonomicrometry for positioning and redundancy) as a key direction. Second, the procedure dictates the modality: epicardial procedures (off-pump CABG, TECAB) favor stereo vision, while intracardiac procedures (mitral annuloplasty, ASD repair, transcatheter valves) require 3DUS with predictive filtering for its latency (2, 45). Third, the control architecture must match sensor latency: 3DUS latency is comparable to the heart’s ejection time and demands predictive control, which remains beneficial even for low-latency force and ECG at high heart rates (32).

## 4 Motion modeling and prediction algorithms

Active compensation is fundamentally a feedforward problem: the robot must move to the predicted future surface position, not the measured present one. Sensing introduces latency of about 12 ms for stereo endoscopy (18) and 50–100 ms for 3DUS (2), and actuation adds more, while the epicardial surface traverses over half its trajectory in the 60 ms ejection phase (6), so any measurement-driven loop lags during this phase. The problem is therefore to estimate the future position over a horizon H from past observations: Nobservations:{z[k−N+1],…,z[k]}⟶z{k+1],…,z[k+H]. Quasi-periodicity (Section 2) makes the motion structured enough for parametric models but non-stationary enough to preclude fixed coefficients. Five paradigms have been pursued: periodic copying, embedding, autoregressive models, Fourier-series Kalman filters, and neural networks, they are summarized in Figure 3.

**Figure 3:**
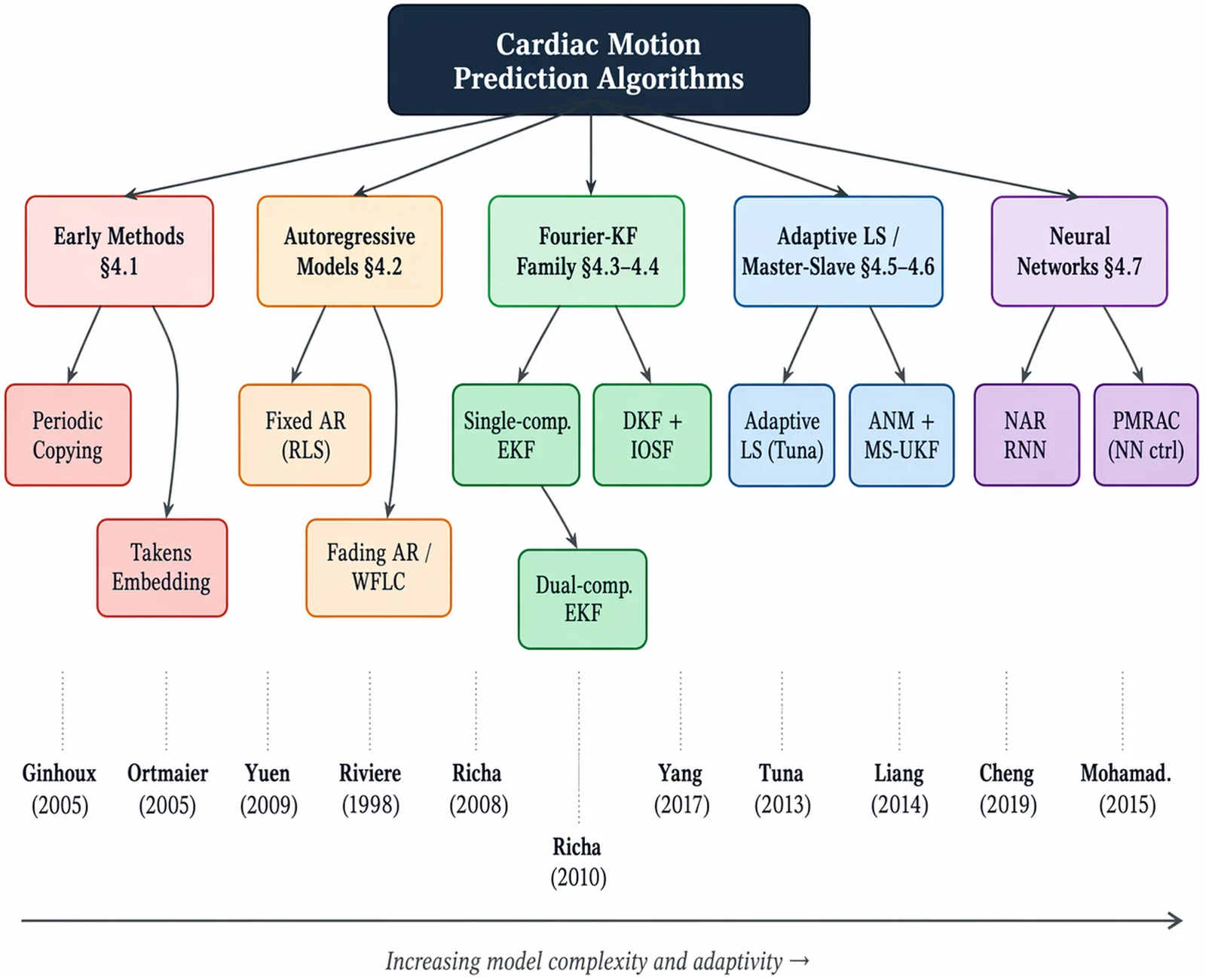
Prediction algorithm taxonomy.

### 4.1 Early approaches: periodic copying and embedding-based prediction

Heartbeat synchronization was introduced by Nakamura et al. [(21), cited in (38)]. Ginhoux et al. [(29), cited in (18)] recorded the heart with a 500-fps camera, modeled respiration as perfectly periodic to separate it from cardiac motion, fitted the cardiac component as a five-harmonic Fourier series offline, and fed it as a feedforward signal to a model predictive controller (MPC). Riviere et al. (17) built the first in-vivo porcine compensation system (500 Hz visual servo with MPC), achieving ∼1.5 mm residual motion and a benchmark for later work. Ortmaier et al. (36) instead used a non-parametric scheme based on Takens’ embedding, forming a delay-coordinate vector $D_{k}=[y[k],y[k-h],\ldots ,y[k-(p-1)h]{]}^{T}$, retrieving the best-matching past vectors, and predicting from a weighted sum of their successors; including ECG and respiratory-pressure signals let the predictor continue through visual loss, and it outperformed an ANN predictor on benchmark sequences. Both early approaches assumed stationary or slowly varying parameters: the Fourier coefficients could not adapt to respiration or autonomic changes, and the embedding method failed when the trajectory departed from past observations. Stoyanov and Yang (4) showed via stability analysis of an AR-based stabilizer that control algorithms were needed for accurate tracking, motivating adaptive parametric models.

### 4.2 Auto-regressive models

The autoregressive (AR) model represents the current position z[k] as a linear combination of its n most recent past values (Equation 4.1):

$$z[k]=\Sigma_{i=1}^{n}\alpha_{i}*z[k-i]+e[k],$$ (4.1)

where the coefficients $\alpha_{i}$ are to be determined, and the term e[k] represents a zero-mean white noise residual (2). At a fixed heart rate, the AR model is equivalent to a harmonic filter bank of order n, and can accurately represent the quasi-periodic motion of the heart. The coefficients $\alpha_{i}$ are estimated via the recursive least squares (RLS) algorithm with forgetting factor λ∈(0,1], which exponentially down-weights older observations to track time-varying cardiac dynamics (20). When λ is set to 1, the algorithm becomes an iterative least squares algorithm with a fixed memory of n observations. When λ is set to a value that is smaller than 1, however, the method becomes the fading memory AR filter, wherein the parameters are updated to reflect only the most recent observations. Franke et al. [(46), cited in Yuen et al. (2)] applied the fading AR filter to motion synchronization in CABG. Riviere et al. (47) developed the related Weighted Fourier Linear Combiner (WFLC) algorithm, in which each component of the Fourier series has an associated gain that can be adapted to allow for synchronization of components of motion at different frequencies.

The study by Yuen et al. (2) performed a definitive evaluation of the performance of the autoregressive models (both fixed and fading memory) relative to the performance of the EKF for the prediction of the motion of the mitral valve annulus from ECG-gated MRI sequences. Monte Carlo simulations were used to evaluate the performance of the algorithms at a variety of noise levels ($\sigma_{R}$ between 0.3 and 3.0 mm), and at heart rate changes of ΔHR between −10 and +10 bpm. EKF outperformed both the fixed AR and fading AR filters in the rate-change simulation: the fixed AR locked on to an average trajectory and degraded as ΔHR increased; the fading AR continuously readjusted to recent noisy data, trading accuracy for adaptability in a regime where the signal variance exceeded the rate-change variation. The WFLC showed EKF-comparable accuracy at small ΔHR but slow convergence at ΔHR>4 bpm. On MIT-BIH Normal Sinus Rhythm records with realistic beat-to-beat variability, only the EKF consistently achieved sub-$\sigma_{R}$ prediction error across all five subjects (2). Despite its relatively poor performance in comparison to the EKF and other models, the autoregressive model is used in various embedded computerized medical systems, most notably force-controlled catheter systems (32).

An alternative implementation of the autoregressive model was presented in Moustris et al. (9) and Moustris and Tzafestas (48) by mapping the input medical image to a canonical stabilized image space via a Strip-Wise Affine Map (SWAM), where the motion synchronization task is reduced to tracking a static reference manifold in canonical coordinates, which is a problem well-suited to linear AR control. Furthermore, such a unification of the concepts of image stabilization and motion synchronization is beneficial in that it enables the creation of higher-level control modes for medical imaging systems that are built upon the synchronization control mode itself.

### 4.3 Fourier series models with extended Kalman filtering

#### 4.3.1 Single-component models and the EKF framework

The Fourier-EKF exploits quasi-periodicity to estimate the model parameters. Richa et al. (22) treated each Fourier coefficient and the fundamental frequency as random walks estimated by an EKF, predicting q steps ahead by evaluating the series at future times (valid for small H) (18, 22); the Fourier observation function is linearized by its Jacobian, making this a true EKF. Convergence from zero initial conditions required about 1.5 respiratory cycles, with $H_{r}=3$ and $H_{c}=5$ harmonics sufficient in vivo (Figure 4). The EKF applied by Yuen et al. (2, 41) to the mitral annulus modeled amplitude and phase per harmonic (Equation 2.2), with state $x(t)=[c(t),r_{i}(t),\omega (t),\phi (t){]}^{T}$ evolving as random walks with covariance Q, initialized by an FFT with a Hamming window; eight harmonics (m=8) with $q_{\omega }=10^{-3}$ rad/s achieved sub-standard-deviation annulus error despite variable heart rates (2).

**Figure 4:**
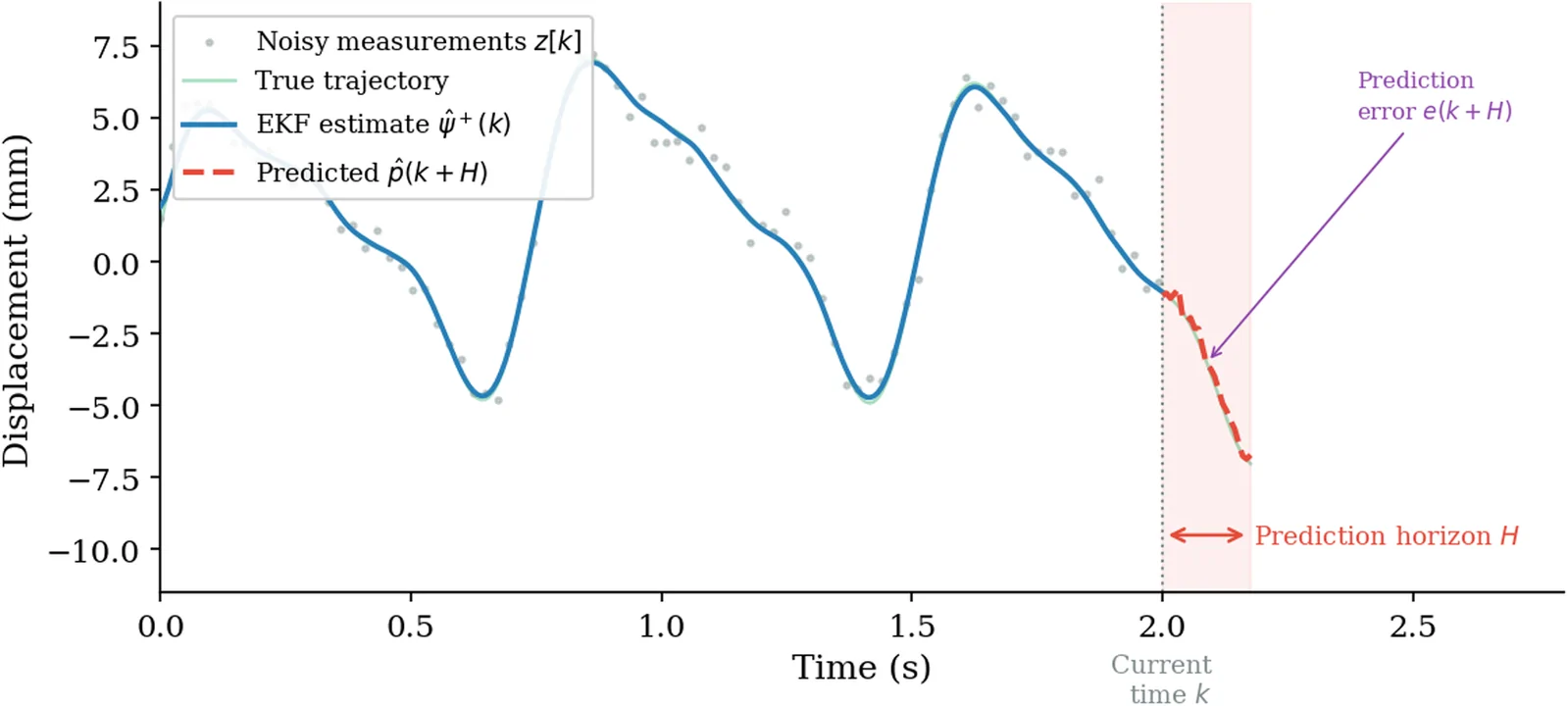
EKF prediction concept.

#### 4.3.2 The dual-component Fourier-EKF

Richa et al. (5) extended the single-component EKF to estimate both respiratory and cardiac components via dual non-stationary Fourier series (Equation 2.1), with shared frequencies across POIs. On three in-vivo datasets (DAVINCI, LIRMM1, LIRMM2) the model achieved 0.73–0.81 mm RMSE at a 0.18 s horizon and 0.80–1.02 mm at 3 s (18). Reducing signal amplitude to 50% caused a transient error increase before re-adaptation, confirming that the random-walk model captures slow drift; a simulated arrhythmia produced high RMSE for about 0.17 s followed by rapid re-convergence, showing recovery from isolated anomalous beats but not sustained arrhythmia. Bachta et al. (28) added a third Fourier series, using ECG and airflow to measure cardiac and respiratory frequencies directly and feeding them to an RLS coefficient estimator; this three-series model outperformed single- and dual-series variants, confirming the physical significance of respiratory–cardiac coupling.

#### 4.3.3 Limitations of the joint EKF

The joint EKF has two limitations. First, linearizing the observation function h(ψ)=p(t,ψ) uses an outdated ω when the frequency changes abruptly, biasing both the mean and covariance (23). Second, its complexity is $O((\gamma \cdot u+2{)}^{3})$ in the number of POIs u, since the covariance matrix is (γ⋅u+2)×(γ⋅u+2). Both are resolved by the Dual Kalman Filter.

### 4.4 The dual Kalman filter and DTVFS

Yang et al. (23) and Liu (37) devised the same solution to the limitations of the joint EKF: separate the estimation of frequency ω and the Fourier series coefficients f, estimating each with its own standard Kalman filter. Given the DTVFS model (Equations 2.1, 2.2), two decoupled sub-filters run in parallel.

$KF_{1}$ estimates the Fourier series coefficients f assuming ω is known. Because ω is treated as a fixed input rather than an estimated state, the observation model $h_{f}(k)$ is linear in f, updated at each time step using the current estimate of ω. A standard (not extended) Kalman filter therefore provides the optimal estimate, eliminating the linearization error that afflicts the joint EKF (23).

$KF_{2}$ estimates ω via the Iterative Optimal Sine Filter (IOSF). The 3D POI trajectory is first projected onto its dominant breathing and heartbeat directions, identified by applying principal component analysis (PCA) to past measurements. The resulting 1D principal-component signals are then passed through a sine FIR filter whose phase is iteratively shifted at each time step to keep the filtered sample on the peak of the fundamental frequency component, allowing instantaneous frequency to be read from three consecutive samples with minimal sensitivity to amplitude noise (23). The estimated ω is fed back to $KF_{1}$ to update $h_{f}(k)$, completing the loop.

Both sub-filters operate in standard Kalman form, removing all linearization error from the non-linear DTVFS model. Results on a real in-vivo da Vinci CABG dataset showed that DKF with IOSF achieved a 50-frame (2 s) prediction RMSE of 0.584 mm compared to 0.681 mm for the joint EKF, while converging rapidly from zero initial coefficient conditions under frequency step changes where the joint EKF diverged into a “self-coincident” state, with a small observation error but severely incorrect internal state estimates, fatal for long-horizon prediction (23). Liu (37) further demonstrated the DKF on in-vivo CABG video sequences, where the DKF tracked the POI through occlusions of up to three seconds and re-converged more quickly than the joint EKF upon visual recovery. Process-to-observation noise variance ratios of $10^{-5}$ to $10^{-4}$ were found to provide stable, well-converged estimates on real da Vinci data (23). The parallel sub-filter architecture is shown in Figure 5.

**Figure 5:**
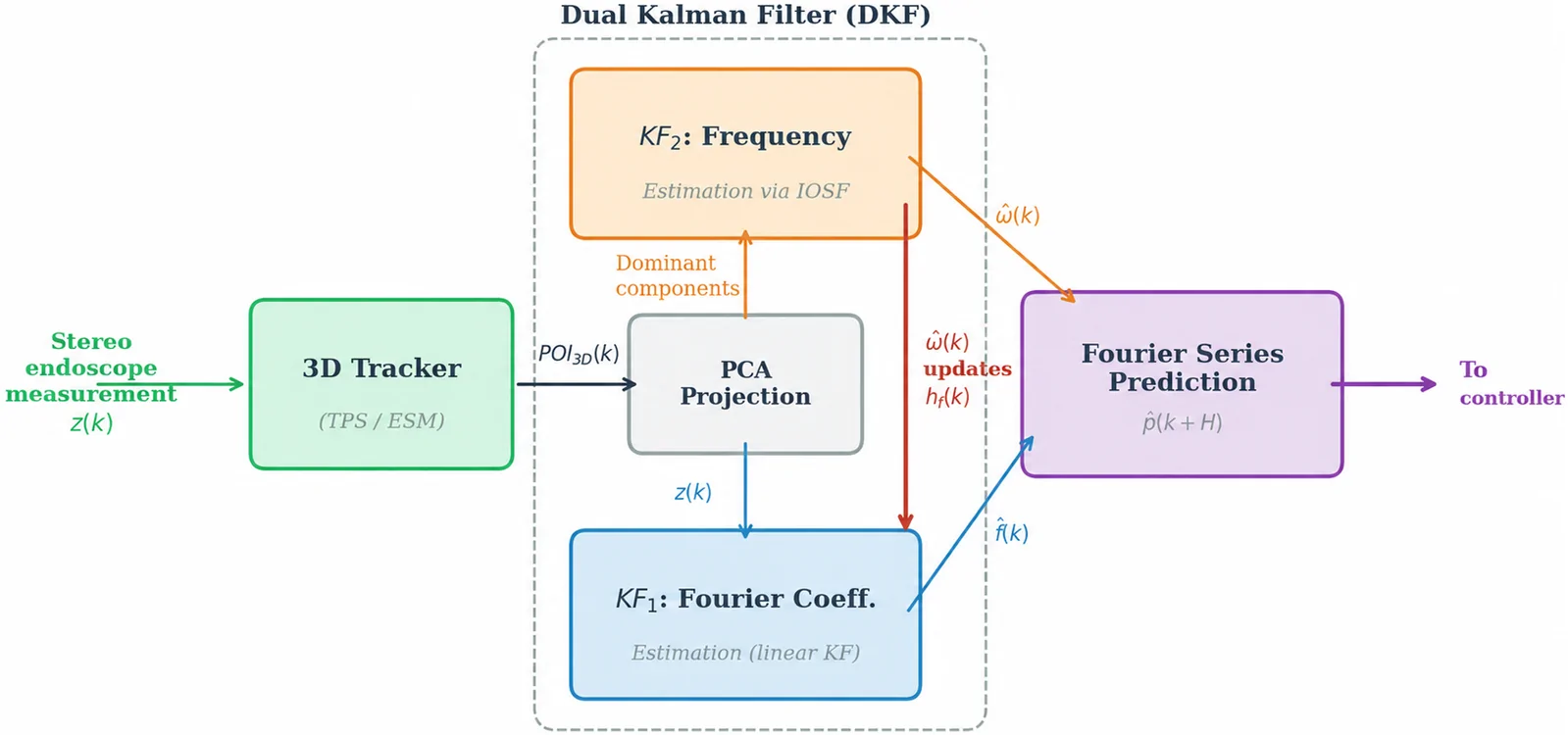
Dual Kalman Filter architecture. Key: $KF_{1}$ and $KF_{2}$ are standard (linear) Kalman filters—no EKF linearization error.

### 4.5 Adaptive least-squares predictors

Tuna et al. (20) approached the prediction problem in a different way: instead of explicitly modeling and fitting a parametric representation of cardiac motion, they noted that the sharp peaks in the Fourier spectrum of heart motion imply that the harmonics do not decay over time, making the trajectory well-approximated by a superposition of periodic signals at constant frequencies. This enables the construction of a linear system (with frequency response matching the Fourier representation of the heart signal, i.e., state-transition matrix with eigenvalues corresponding to the dominant spectral peaks) without explicit frequency estimation. The one-step prediction scheme estimates the state-transition matrix U (mapping the observation at time n to a predictor of the observation at n+1) via RLS with exponential windowing.

The generalized predictor developed in (20) independently fits each point of the M-step horizon using a matrix V composed of sub-matrices $\phi_{0,i}(i=1,\ldots ,M)$, and can be seen as an extension of the one-step algorithm that works with an equivalent RLS-type update for U with introduced delays to resolve the causality constraint, as given in Equation 4.2:

$$\hat{z}[n+M]=V*\phi [n]=\Sigma_{i=1}^{M}\Phi_{0,i}*\phi_{i}[n]$$ (4.2)

The gain in computational efficiency over a bank of M one-step predictors is in sharing the input covariance update across all horizon points, cutting complexity down by a factor of M (20). Experimental validation on three in-vivo calf datasets using an artificial pacemaker to sweep heart rate within a controlled range showed that the generalized adaptive predictor outperformed the one-step predictor in terms of RMS position error and was competitive with the EKF predictor, with the relative advantage varying by dataset. A 16th-order one-step estimator was used, which could only estimate one prediction point, while hardware experiments employed a generalized estimator of 10th order predicting 4 points over a 25 ms horizon. The main advantage over the Fourier-EKF is that no explicit frequency estimation is required, so there is no convergence transient at filter initialization.

Vrooijink et al. (45) took a scheduled-prediction approach for transcatheter aortic valve implantation: instead of fitting a model during operation, they extracted aortic heart valve (AHV) motion profile from pre-operative 2D ultrasound images, yielding a patient-specific model encoded as the a priori reference trajectory $r_{AHV}$ in a constrained MPC formulation (Kalman filtering out measurement noise). The MPC then minimizes a quadratic cost function subject to constraints on tip velocity and tip deflection, using the ultrasound signal after processing by an online Kalman filter to provide feedback on tip position. This pre-operative motion model approach allows accurate anticipation of the AHV trajectory, avoiding transient convergence effects associated with online parametric methods but at the cost of requiring a pre-operative data acquisition step specific to each patient; furthermore, it may be less robust to heart rate variability during surgery.

The semi-autonomous impedance control approach of Cheng et al. (49) represents the minimal-model extreme of the prediction design space, with the frequency band of physiological motion being the only required a priori information, and no parametric model fitted. A frequency selective impedance controller rejects the oscillatory component of the robot-heart interaction force within a defined frequency band, passing through the non-oscillatory (task specific) component to the surgeon as haptic feedback. The approach trades prediction accuracy (and hence compensation fidelity) for robustness to model mismatch and implementation simplicity, with an eye toward applicability in situations where the sensor does not provide (near) exact information on frequency content.

### 4.6 Master-slave Kalman filter for nonlinear and uncertain motion

Liang et al. (3) addressed the common limitation of Fourier-based models, which is their inability to capture the deterministic nonlinearity of cardio-respiratory coupling, with the Adaptive Nonlinear Model (ANM), decomposing motion into three components (Equation 4.3):

$$X(t)=X_{c}(t)+X_{r}(t)+X_{m}(t),$$ (4.3)

with cardiac $X_{c}$, respiratory $X_{r}$, and nonlinear coupling $X_{m}(t)=X_{c}\cdot X_{r}$, each linear component a time-varying cosine Fourier series (Equations 4.4a–4.4c): ?>

$$X_{c}(t)=\Sigma_{k=1}^{Nc}a_{k}^{c}\cos\;(2\pi *f_{k}^{c}*t+\phi_{k}^{c}),$$ (4.4a)

$$X_{r}(t)=\Sigma_{l=1}^{Nr}a_{l}^{r}\cos\;(2\pi *f_{l}^{r}*t+\phi_{l}^{r}),$$ (4.4b)

$$X_{m}(t)=\Sigma_{k,l}a_{kl}^{m}\cos\;(2\pi *f_{kl}^{m}*t+\phi_{kl}^{m})c)$$ (4.4c)

with coupling terms $f_{kl}^{m}=f_{k}^{c}+f_{l}^{r}$ and $\phi_{kl}^{m}=\phi_{k}^{c}+\phi_{l}^{r}$ representing the sum-frequency components. A Master UKF propagates all amplitudes, frequencies, and phases as random walks using sigma points to avoid linearization, while a Slave UKF inflates its process-noise covariance $Q_{vk}$ on anomalous-beat residuals (3). On two in-vivo calf datasets (sonomicrometry) ANM achieved 3D RMSE of 20.1 and 22.4μm, vs. 227.4 (AR), 131.5 (FS-EKF), 60.8 (TQN), and 57.4μm (FQN) (3). Crucially, it was also tested on two MIT-BIH arrhythmia ECG recordings (106m, PVC; 203m, atrial flutter/fibrillation), achieving RMSE of 18.1 and 15.7μV, this makes it the only reviewed method validated on actual clinical arrhythmia, with the Slave UKF inflating $Q_{vk}$ on arrhythmic beats and recovering once sinus rhythm returns, giving graceful degradation rather than phase loss. A comparison across methods is shown in Figure 6.

**Figure 6:**
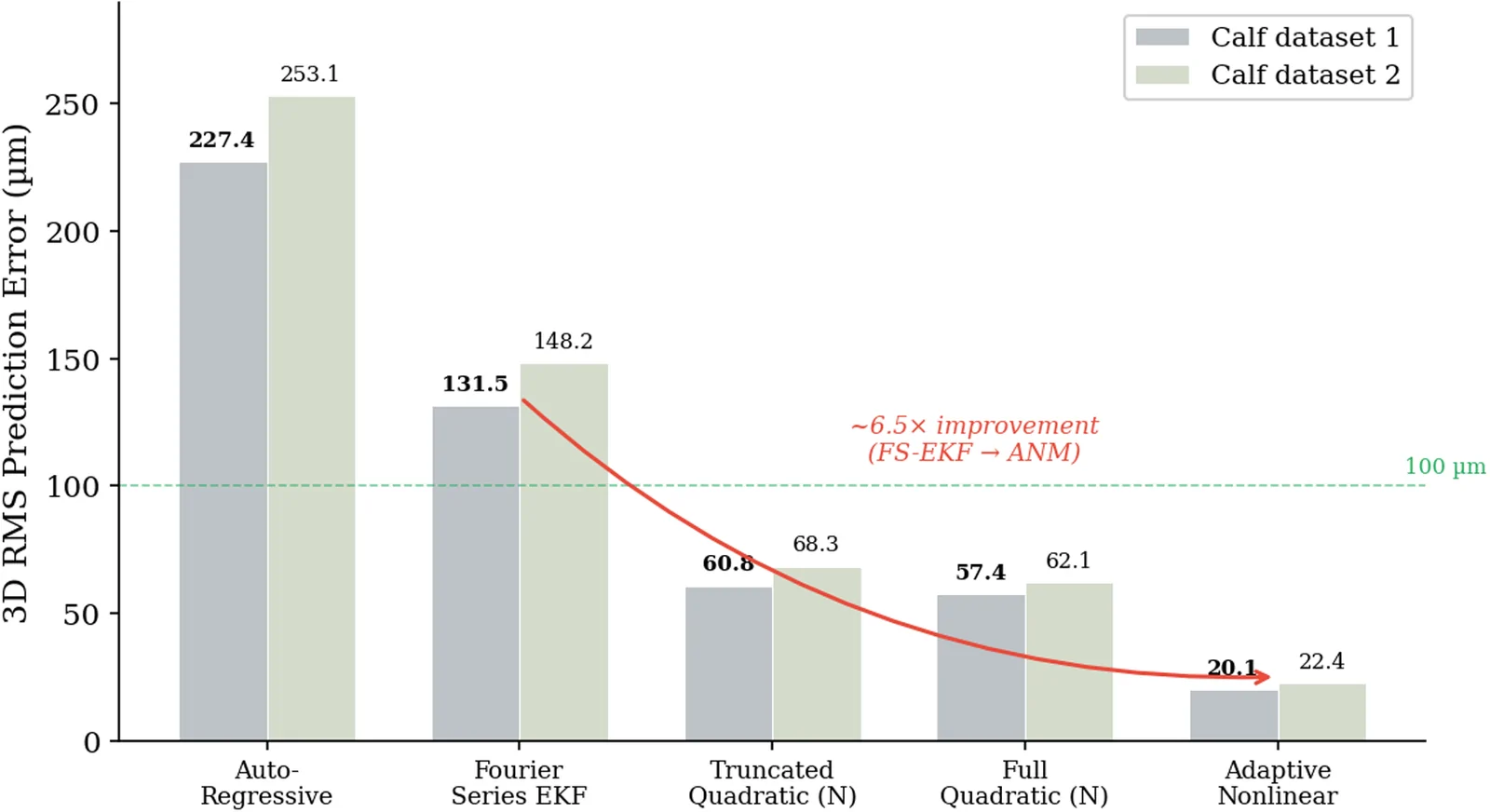
Prediction accuracy comparison.

### 4.7 Neural network and machine learning approaches

The most recent shift is toward recurrent neural networks (RNNs), reflecting the inability of parametric models to capture the full nonlinear dynamics across patients and conditions. Ortmaier et al. (36) included an early neural predictor that was outperformed by the embedding method, a negative result that delayed NN research for over a decade until larger datasets and deep recurrent architectures renewed interest. Cheng and Tavakoli (50) introduced a Nonlinear AutoRegressive (NAR) RNN for ultrasound-guided surgery to counter the ∼160 ms imaging latency, using a closed-loop two-hidden-layer structure with output feedback so that, from input $x(n)=[x(n-1),\ldots ,x(n-L){]}^{T}$, the network learns the mapping in Equation 4.5:

$$\hat{x}(n+D)=f_{NN}(x(n),x(n-1),\ldots ,x(n-L)),$$ (4.5)

with D the compensated latency (50). Trained on ten 1000-s sequences (75% train, 25% test) at fixed and variable rates, the NAR network achieved about 60% lower MAE and RMSE than the EKF at a 160 ms horizon on both datasets. Mohamadipanah et al. (8) instead used neural networks as the adaptive plant and controller of a predictive model-reference adaptive controller (PMRAC), continuously retraining on a rolling 20-sample window to follow drifting waveform morphology and achieving under 0.15 mm control error on a real human heart dataset. The systematic mapping study of Mohd Fua’ad et al. (11) surveyed 23 papers and identified the main challenges as the train-to-deployment generalization gap, the lack of public surgical motion datasets, and ethical concerns over patient data, which is consistent with the central difficulty that a network trained on ventilated CABG patients may not transfer to spontaneously breathing mitral-repair patients, whose frequency content, amplitude, and coupling differ substantially. Table 2 compares all methods across horizon, accuracy, arrhythmia robustness, and validation context; because validation substrates and error units differ, the table maps the methodological landscape rather than ranking methods on a common scale.

## 5 Control architectures for motion compensation

The prediction module of Section 4 turns the sensing of Section 3 into a reference trajectory; the control architecture turns that reference into robot commands. It must track the predicted POI within the tool error budget, reject the residual error from model mismatch, arrhythmia, and noise, and, for contact tasks, regulate force to avoid tissue injury, all while remaining stable on the teleoperated platform against the non-passive beating heart and any communication delay. Four general architectures exist: position control (leaving disturbance rejection to the predictor); vision-feedback control (generalized predictive or repetitive control of observed periodic motion); a Kalman Active Observer within an MPC loop (joint force control and autonomous compensation); and shared or teleoperated control that keeps the surgeon in the loop via a stabilized display or bilateral haptic interface.

### 5.1 Feedforward position control

The simplest complete motion compensation system places the predictor in the feedforward path and employs a stiff inner position servo loop to track the predicted position of the POI. The predictor calculates the desired position of the POI in the future $\hat{\;p}(t+H)$ and that position is fed to the robot controller.

The inner loop of the position control system was developed by Ortmaier and Hirzinger in (51) and (52). A key practical challenge in surgical robots is that individual joints exhibit different rise times $T_{i}\neq T_{r}$, causing the Cartesian time-response to become working-position dependent and coupling the Cartesian degrees of freedom. To account for this behavior, the authors developed a self-adjusting lead-lag controller that automatically set the gain (K) and time constant ($T_{1}$) of the controller to balance the open-loop response of the robot system. This controller was validated on a 2-DOF SCARA robot and was shown to reduce the error of the Cartesian cross-coupling motion by 61% compared to using a lead-lag controller with fixed gains.

Ortmaier et al. (36) implemented this type of control system within the context of beating-heart surgery, along with the tracking and prediction algorithms presented in section 4. The overall system feeds the predicted heart surface trajectory into the robot control block as a Cartesian reference. An inner position servo system based on the gain-scheduling algorithm of Ortmaier and Hirzinger (51) is used to track this desired position, while the surgeon’s telemanipulator controls are also superimposed upon this position control. The MIS constraint on the fulcrum point of the heart is incorporated into the control law for the Cartesian system, and a self-adapting force control law reduces injury risk during unexpected contact. These techniques were validated on five operations that performed in-vivo on beating hearts. As a result, the system achieved a prediction accuracy of approximately 2 px (∼0.4 mm), a tenfold reduction in the ∼6–8 mm uncompensated residual motion (36).

Riviere et al. (17) were the first to implement such a system in-vivo on a porcine heart. The authors used a 500 Hz system based on visual servoing and model predictive control, which achieved a motion residual of approximately 1.5 mm of motion.

A limitation of the feedforward control system is that there is no means of correcting any error between the current position of the surgical instrument and the predicted position of that instrument. The error between the current position of the instrument is defined as the tracking error: $e(t)=p(t)-\hat{\;p}(t+H)$. This error can be introduced by spectral components beyond the model order, arrhythmic beats that violate quasi-periodicity, and latency mismatch between prediction horizon and loop delay.

### 5.2 Visual-servo predictive control

Ginhoux et al. (29) treated physiological motion as a measurable disturbance to a static reference and used a Generalized Predictive Controller (GPC) to cancel it; such disturbance-rejection architectures are robust to predictor misfit because the feedback loop corrects residual error.

#### 5.2.1 R-GPC for respiratory motion

Because ventilated respiratory motion is strictly periodic, it suits iterative learning control. Ginhoux et al. encoded a periodic disturbance of period $N_{s}$ in a modified ARIMAX operator with forgetting factor ρ∈(0,1) and split the control into surgeon-reference and learned-disturbance components to avoid overshoot (29), reducing respiratory-motion variance by 93% in porcine in-vivo tests.

#### 5.2.2 Adaptive GPC for cardiac motion

Cardiac motion cannot use repetitive control because the cardiac and respiratory components are non-harmonic (irrational, rate-varying ratio). Ginhoux et al. (29) first separate the components (equivalent to the EKF Fourier decomposition), then predict disturbance samples $\hat{d}(k+1),\ldots ,\hat{d}(k+H)$ over the GPC horizon, with cost function in Equation 5.1:

$$J=\Sigma_{\;j=1}^{H_{p}}[y(k+j)-w(k+j)+\hat{d}(k+j){]}^{2}+\lambda *\Sigma_{\;j=0}^{H_{u}-1}\Delta u(k+j{)}^{2},$$ (5.1)

where w is the surgeon’s reference, $\hat{d}$ the predicted disturbance, $H_{p}$ and $H_{u}\leq H_{p}$ the prediction and control horizons, and λ the control weight. Including the disturbance lets the GPC anticipate and pre-compensate it, reducing cardiac-motion variance by 80% at 500 Hz (29); the method was also implemented on the Aesop robot by Riviere et al. (17). Moustris and Tzafestas (9) further showed that recasting the visual servo as a stabilized control system simultaneously stabilizes both the surgeon’s image and the instrument motion.

### 5.3 Force-controlled architectures

#### 5.3.1 The viscoelastic interaction model as control basis

For contact tasks, the main control variable of the robot along with position is the force at the tool tip. The first step to designing an appropriate force controller for a robotic surgical system is to select an interaction model that describes the soft tissue realistically. The following discussion starts with linear viscoelastic models and leads to the nonlinear Hunt-Crossley model, following the direction of research performed by the LIRMM group.

Liu et al. (35) performed the first study on force control for robotic-assisted beating heart surgery using a realistic model of viscoelastic tissue. Their experiments used the D2M2 robot to perform in-vitro tests on soft tissue to determine the parameters of four different models of viscoelasticity: Maxwell, Kelvin-Voigt, Kelvin-Boltzmann, and a fractional-derivative model. The Kelvin-Boltzmann model, which is a spring and damper in parallel, connected in series with another spring is modeled as in Equation 5.2:

$$\begin{array}{rl} & F(t)=\gamma x(t)+\beta \dot{x}(t)-\alpha \dot{F}(t),\;\alpha =\frac{b}{x_{1}+x_{2}},\\ & \;\beta =\frac{bk_{2}}{k_{1}+k_{2}},\;\gamma =\frac{k_{1}k_{2}}{k_{1}+k_{2}} \end{array}$$ (5.2)

Based on this model, Moreira et al. (34) designed a force controller for the robotic system, proving the stability of the resulting system analytically. Additionally, they simulated the system using in-vivo daVinci heart data to prove the success of the system. Hence, this was the first attempt at designing a force controller for robotic systems based on a viscoelastic interaction model.

Liu (37) noted a problem in all of the linear viscoelastic models: they predicted a symmetric hysteresis loop in the relationship between loading and unloading of the soft tissue; in reality, the loop is asymmetric. The nonlinear Hunt-Crossley model models soft tissue by replacing the linear spring and damper of the Kelvin-Voigt model with a nonlinear one, giving Equation 5.3:

$$F(t)=K*x^{\beta }(t)+\lambda *x^{\beta }(t)*\dot{x}(t),$$ (5.3)

where β∈[1.1,1.3] for soft tissue, and λ is the nonlinear damping coefficient (37). Tests on a lamb’s heart in vitro using a RAVEN-II surgical robot indicated that the Hunt-Crossley model produced a relationship between loading and unloading that was both asymmetric and physically correct (F=0 when x is 0, during penetration and unpenetrating the tissue), whereas the Kelvin-Boltzmann model could not achieve either property. The Hunt-Crossley model can be linearized around an operating point $x_{0}$ to obtain a state-space plant model suitable for observer-based control design (37).

The discretized plant model, with state vector $x_{r,k}$ containing force, force rate, and delayed input, serves as the basis for the AOB design described in Section 5.3.2 (37). Simulation and ex-vivo experiments using the Hunt-Crossley model with an AOB force controller showed that this controller outperformed those using the elastic, Kelvin-Voigt, and Kelvin-Boltzmann models (37). Hence, Liu’s model both reaffirmed the findings of Moreira et al. (34) regarding the importance of nonlinearity in the control system, while additionally adding justification for using the nonlinear model due to its physical correctness.

#### 5.3.2 The Kalman active observer

Cortesão and Poignet (42) introduced the Active Observer (AOB) to remove the estimation error a standard Kalman filter injects into state feedback: an extra “active” state $p(k)=L\hat{e}(k)$ is appended as a zero-mean random walk, and tuning its covariance $Q_{p}$ imposes the desired closed-loop dynamics $G_{cl}(z)$ regardless of tissue stiffness (37, 42). The same work used two independent AOBs, a first-order AOB for force and a second-order AOB for physiological-motion compensation requiring no a priori motion model, which on a 7-DOF WAM arm with an ex-vivo porcine heart reduced peak-to-peak physiological force by 60%–72% (43) and was robust to robot–tissue stiffness mismatch, since the active state absorbs compensation errors before they reach the force loop.

#### 5.3.3 Cascade MPC-AOB architecture

While this dual Active Observer architecture of (42) achieves autonomous motion compensation without any model of the motion of the cardiac tissue, the performance of the system is limited by the bandwidth of the Kalman filter. Dominici and Cortesão in (53) propose an architecture that cascades two control loops: an inner AOB loop, and an outer MPC loop. The motivation for this cascade structure is that it allows for the AOB to absorb the uncertainty in the model of the system (due to the stiffness differences between robot and tissue, for instance), while the MPC outer loop can use a model of the stabilized system to compensate for physiological motion.

In the MPC outer loop, the model of the system in state space form is used to predict the future outputs of the system over a prediction horizon $H_{p}$ (Equation 5.4):

$${\tilde{X}}_{k}=\Psi {\hat{x}}_{k}+\Upsilon u_{k-1}+\Theta *\Delta {\tilde{U}}_{k},$$ (5.4)

where ${\tilde{X}}_{k}=[{\tilde{x}}_{k+1}\ldots ,{\tilde{x}}_{k+H_{p}}{]}^{T}$ is the future state vector to be predicted, $\Psi =[A,A^{2},\ldots ,A^{H_{p}}{]}^{T}$ is a matrix that encodes the free response of the system to initial conditions, Υ is a matrix that describes the effect of the most recent input to the system, Θ is a Toeplitz matrix whose elements are the step responses of the system, and $\Delta {\tilde{U}}_{k}=[\Delta u_{k},\ldots ,\Delta u_{k+H_{u-1}}{]}^{T}$ is a vector of control changes to be applied over a control horizon of $H_{u}\leq H_{p}$ (53). A cost function (Equation 5.5) is used to find the control changes:

$$W_{k}=\delta \Sigma_{i=1}^{H_{p}}[{\tilde{y}}_{k+i}-F_{d}{]}^{2}+\lambda \Sigma_{i=0}^{H_{u}-1}[\Delta {\tilde{u}}_{k+i}{]}^{2}$$ (5.5)

This cost function is minimized at each sample to compute $\Delta {\tilde{U}}_{k}$ ; only the first element $\Delta u_{k}|i=0$ is applied to the AOB inner loop (receding horizon principle). The matrices δ and λ are diagonal weighting matrices for tracking error and control effort respectively: increasing λ/δ reduces control activity at the cost of slower disturbance rejection (53).

The advantages of using this cascade structure of MPC and AOB are the robustness of the system to stiffness mismatches between the robot and the tissue being manipulated. Because the AOB compensates for physiological motion over the time steps of the control horizon, the model of the system used by the MPC is the same as that used for the AOB, allowing it to accurately compensate for physiological motion even in the presence of stiffness mismatches of the two systems. The architecture was tested on three tissue surrogates of different stiffness (pillow 375 N/m, heart 810 N/m, sponge 1,900 N/m) yet it maintained an accuracy of within ±0.2 N in peak-to-peak force for all three tissue types. A 3 degree-of freedom robot with an ex-vivo heart was able to simultaneously reproduce the physiological cardiac and respiratory motions of the heart.

#### 5.3.4 Iterative learning control for periodic disturbances

Iterative learning control was proposed by Cagneau et al. [(54), cited in (53)] as a model-independent outer loop that learns the periodic disturbance over time. Its disadvantage for cardiac surgery is the assumption of a known, stable disturbance period, which is violated by beat-to-beat variation and respiratory–cardiac coupling (though satisfied for respiration alone); the cascade MPC-AOB architecture is therefore more effective for cardiac applications.

### 5.4 Shared control and the canonical stabilization framework

Full autonomy raises safety and ethical challenges qualitatively different from standard telerobotics (17): a fully autonomous robot would move to the wrong place on a mispredicted arrhythmic beat with no surgeon correction. Shared control preserves the surgeon’s intent while adding robotic synchronization. Moustris et al. (55) established a canonical framework using the Strip-Wise Affine Map (SWAM), later used in (9), mapping the physical workspace $W_{p}$ (where the heart beats) to a canonical world $W_{c}$ (where it is stationary) via $\Phi \;:\;W_{c}\to W_{p}$. The framework unifies three functions:

(i) **Mechanical synchronization:** the slave robot holds a fixed pose in $W_{c}$, so it physically tracks the heart without surgeon input.

(ii) **Image stabilization:** the endoscopic image is rendered in $W_{c}$, so the heart appears stationary to the surgeon.

(iii) **Active assistance:** a spring-mass-damper attractor in $W_{c}$ guides the tool toward the desired trajectory without removing surgeon intent, generating an assistive force proportional to the canonical master–tool separation (Equation 5.6):

$$F_{assist}=-k_{s}{(}^{c}y_{m}{-}^{c}y_{r})-b_{s}(\dot{{}^{c}y_{m}}-\dot{{}^{c}y_{r}}),$$ (5.6)

with spring and damping terms $k_{s}$ and $b_{s}$ (9). Experiments with a trained surgeon showed a 41% reduction in mean tracking error and 38% in RMSE at 18 bpm (55) (Figure 7). Shared control is also safer: the surgeon stays in the loop and the assistive force is bounded by the spring constant, so the robot cannot move to a wrong position without surgeon intervention.

**Figure 7:**
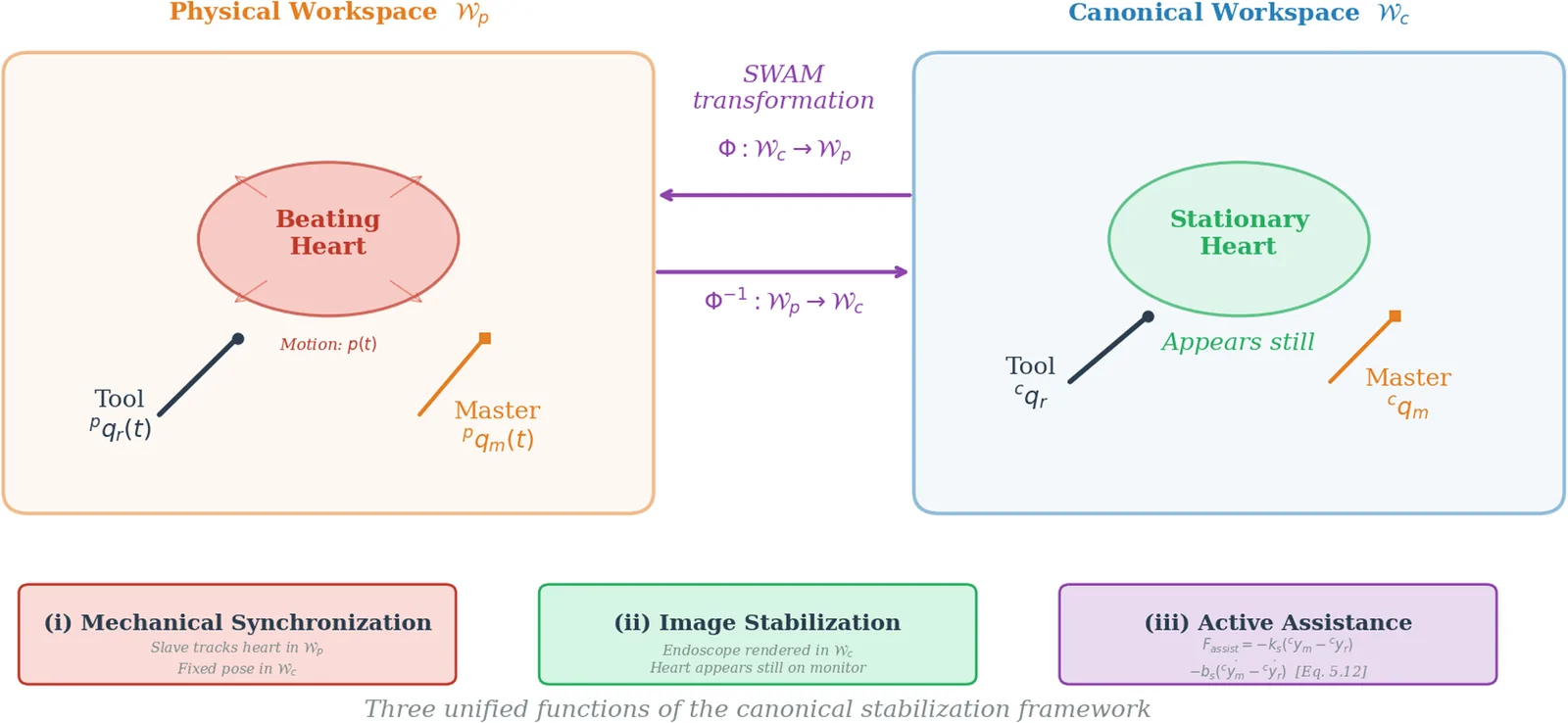
Canonical stabilization framework (SWAM).

### 5.5 Haptic teleoperation with passivity and motion compensation

Operating a remote console over a communication channel raises two stability problems: small channel delays can destabilize the bilateral loop (37), and the standard passivity assumption is violated because the beating heart is an active environment that injects energy.

#### 5.5.1 The active environment problem

Liu (37) analyzed the interaction impedance E(ω) seen by the slave robot in contact with the beating heart and showed, by simulating a porcine heart and modeling the surgeon’s hand, that Re(E(ω))<0 at all cardiac frequencies, i.e., the heart is non-passive. Since classical bilateral-teleoperation stability theory assumes passivity, the three existing architectures were instead analyzed with Llewellyn’s absolute-stability criteria (which do not require a passive environment), identifying the P-PF architecture as the best choice for surgery (37).

#### 5.5.2 Wave variable compensation for delayed teleoperation

For systems with non-negligible communication delay, the Wave Variable Transformation (WVT) is the established method for passivating the delayed communication channel. However, it has been found that the classical method of using wave variables has an undesirable bias in the tracking of both position and force of the robot (37).

The solution proposed by Guo et al. (56) is to modify the wave variable transformation by including two compensation terms that counter the effects of the time delay in the communication channel. The compensation terms are defined in Equations 5.7a and 5.7b:

$$\Delta_{u}(t)=v_{m}(t-T)-v_{s}(t),$$ (5.7a)

$$\Delta_{v}(t)=u_{m}(t)-u_{s}(t-T),$$ (5.7b)

where the terms $u_{m}$ and $v_{m}$ are the wave variables of the master robot, and the terms $u_{s}$ and $v_{s}$ are the wave variables of the slave robot. The compensation terms ensure that the slave tracks the master in both position and force with delay equal to the minimum one-way channel delay T, eliminating the steady-state bias of classical wave variables (56). The introduction of these terms, however, has the potential to introduce energy into the teleoperation system and violate the passivity condition. In order to ensure passivity for the compensated system, energy reservoir regulators $E_{s}(t)$ and $E_{m}(t)$ are constructed and the compensation terms are modulated by an exponential saturation based on the stored energy (Equations 5.8a and 5.8b):

$$\Delta u^{\prime }(t)=\alpha *(1-\exp\;(-\beta *E_{s}(t)))*(v_{m}(t-T)-v_{s}(t)),$$ (5.8a)

$$\Delta_{v}^{\prime }(t)=\alpha *(1-\exp\;(-\beta *E_{m}(t)))*(u_{m}(t)-u_{s}(t-T))b)$$ (5.8b)

When the energy stored in the reservoir is zero, the compensation terms reduce to their classical wave variable method counterparts (ensuring passivity), and as the reservoir fills with energy, the compensation terms are activated (37). These methods were tested using two Omega-7 robotic devices, where the master and slave were connected through a communication channel that simulated the delay introduced through ZigBee wireless communication. The simulations indicated that the stability of the bilateral teleoperation system was maintained, and that the bias in position and force tracking was eliminated. Furthermore, the method was extended by Guo et al. (56) to allow force and position scaling between the master and slave devices for applications in neurosurgery: velocity/position and force scalings were designed within the introduced wave variable compensation (WVC) framework with guaranteed passivity, enabling applications where the slave operates at a different force or position scale from the master.

The motion synchronization introduced in Section 4 has the potential to reduce the impact of the active environment of the heart. Specifically, by reducing the components of the heart motion that are reflected in the motion of the slave robot, the real part of the interaction impedance is reduced, which brings the system closer to the condition of passivity for the environment. Good motion prediction is therefore not only a tracking accuracy requirement but a stability prerequisite for force-feedback teleoperation of beating heart surgery, as reflected in the architecture of Figure 8.

**Figure 8:**
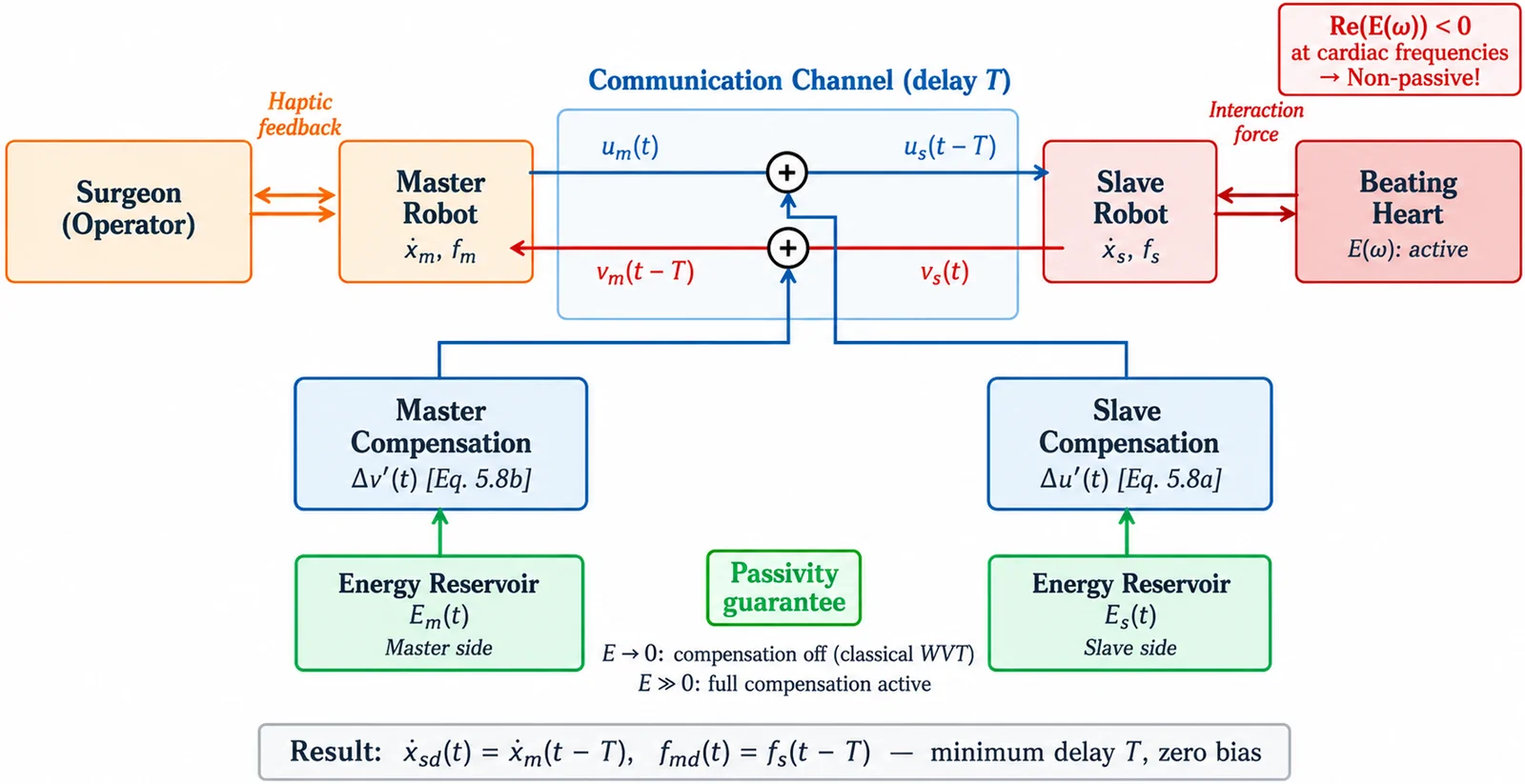
Bilateral teleoperation with wave variable compensation.

## 6 Clinical translation, open challenges, and future directions

Clinically, active closed-loop compensation would make two kinds of difference. First, it would improve existing off-pump CABG: sub-millimeter tool-to-tissue tracking on the beating surface supports more precise, reproducible anastomoses and could reduce conversions to on-pump surgery, extending the benefits of avoiding cardiopulmonary bypass to patients currently considered too complex. Second, it would broaden robotic surgery to intracardiac and structural beating-heart procedures that motion currently makes impractical such as mitral valve repair (6) and off-pump neochordae implantation (where operating on the beating heart additionally lets the surgeon tune chord length and assess leaflet coaptation under physiological load), as well as arrhythmia and transcatheter interventions (45) that demand precise tracking of fast-moving targets.

Despite two decades of progress, active closed-loop algorithmic motion compensation has not yet crossed into human clinical trials, although image-stabilization, vision-based tracking, and mechanically-stabilized robotic approaches have reached the clinic (Section 6.2); this section examines the translation arc, the systems that reached clinical environments, the remaining barriers, and the most promising research directions.

### 6.1 From proof of concept to clinical pipeline

#### 6.1.1 The mechanical stabilizer era and its residual motion problem

Before motion compensation emerged as a discipline, mechanical stabilizers were already being assessed for beating-heart procedures. Gründeman et al. (30) used the Endo-Starfish positioner and Endo-Octopus stabilizer with the Da Vinci telemanipulator to restrain the coronary target to roughly 1×1 mm, sufficient to complete TECAB anastomoses in a porcine model. Lemma et al. (31) then performed the first quantitative measurement of residual motion under three commercial stabilizers, finding that the motion persisting after stabilization was primarily respiratory and was not suppressed by the stabilizers. These studies established the requirement that motion compensation address what mechanical stabilization cannot eliminate.

#### 6.1.2 First robotic synchronization—Nakamura et al. (2001)

The first robotic heartbeat synchronization was reported by Nakamura et al. (21), who used a 955-fps stereo camera and a 4-DOF slave robot to follow a laser point on the beating heart while the surgeon at a PHANToM console perceived a stationary target. Cardiac and respiratory components were identified with a sixth-order autoregressive model, achieving a maximum deviation of about 0.5 mm in laboratory tests. This established the concepts of heart–robot synchronization, image-stabilized surgical display, and shared human–robot control that recur throughout the field.

#### 6.1.3 Intracardiac extension—the Harvard Howe Group (2008–2013)

The most sustained pre-clinical program came from the Harvard Howe group, which progressed from epicardial compensation to robotic intracardiac surgery (Table 3). Their 1-DOF, voice-coil motion-compensation instrument (MCI) tracked the mitral annulus under 3DUS and cut anchor failure rates by about 60% vs. a solid instrument (57); a follow-up established 1.15±0.004 mm RMSE with EKF prediction robust to 39–89 ms delay and 4.0 bpm heart-rate variability (2). The 2013 in-vivo “flashlight” system reached 0.8–1.0 mm RMSE across two porcine trials vs. 3.2 mm for manual tracking (a 70% improvement), with the 68 ms 3DUS delay compensated by the EKF (6). Kesner and Howe (7) then extended the approach to a transvascular catheter, achieving 1.0 mm RMSE in vivo vs. over 8 mm uncompensated; the first robotic catheter capable of compensating fast intracardiac motion.

#### 6.1.4 Clinical robotic CABG—the Da Vinci TECAB program

In parallel with the laboratory research programs, clinical robotic cardiac surgery advanced along a separate but related axis: the maturation of the Da Vinci robotic TECAB program. Balkhy published a detailed study of the developments of what is essentially a high volume program for performing beating heart TECAB procedures (58). The program utilized the Endowrist Stabilizer (Intuitive Surgical) to locally stabilize the heart in the targeted vessel within the Da Vinci workspace without the need for an additional mechanical stabilizer for the heart, and removing the associated hemodynamic compromise of full cardiac displacement. Additionally, the C-Port Flex A distal anastomotic connector (Aesculap) was used in some of the procedures performed with the program to reduce the technical intracorporeal suturing step; however, these surgical connectors were eventually discontinued, which limited broader TECAB adoption. As for outcomes, Balkhy reported that 75% of patients required no opioid analgesia after the initial week following surgery, that the mean time to return to work and physical activity was 14 days after undergoing the procedure, and that patency rates at 10 years were maintained in a series of 570 patients who underwent the procedure (58).

An additional related study was published by Lutz et al. (15), who detailed their experience with robotic minimally invasive direct coronary artery bypass (MIDCAB) procedures over a period of 20 years. They identified the key procedural principles that reduce the learning curve: rigorous patient selection, concentration of early experience in one or two surgeons to maximize proficiency, engagement of the whole operating team, flexibility in anastomotic technique, and patience during the learning phase. Additionally, the recommendation that robotic surgeons gain experience performing the left internal thoracic artery (LITA) harvest procedure (which can be performed endoscopically) before performing the anastomoses was found to be effective; the proficiency in performing the procedure was acquired after 10 to 50 cases. Furthermore, considerable improvements in the times required to perform the procedure and the rates of complications from the procedure were documented after the 10th case performed by each surgeon.

Baudo and Torregrossa (14) synthesized the global literature on robotic CABG, encompassing 11,135 minimally invasive CABG procedures predominantly from Central Europe, the US East Coast and South, and East Asia. Pooled outcomes showed that survival rates after one year of the procedures were 95.2%, survival rates after five years were 83.2%, a 4.7% intraoperative conversion rate (95% CI: 1.6–9.1%), and 93.4% freedom from major adverse cardiac events.

#### 6.1.5 Live clinical motion tracking—Stenmark et al. (2022)

Most recently, Stenmark et al. (44) deployed a vision-based 3D tracking system using ArUco markers on standard forceps in two live pediatric open-heart surgeries, achieving 1.9–2.6 mm accuracy at 30–50 ms per frame on a standard laptop with negligible surgeon inconvenience. This is the first documented use of computer-vision motion tracking in a live human cardiac operating theater, demonstrating that the pre-clinical-to-clinical step for tracking is achievable with modest engineering.

### 6.2 Comparative summary of validated systems

Table 3 summarizes the principal systems across target procedure, guidance modality, validation level, and key outcome; because validation models and outcome definitions differ, it contextualizes translational maturity rather than ranking performance. A clear pattern emerges: the laboratory programs (Nakamura, Yuen, Kesner) progressed through in-vivo porcine validation but have not entered human trials with active robotic compensation; the clinical CABG programs (Balkhy, Baudo, Lutz) achieved large-scale human use but rely on mechanical stabilization; and Stenmark et al. is the only motion-tracking technology deployed in live human surgery, in a tracking rather than compensation role. This gap, active algorithmic compensation validated in animals but not yet in human trials, is the central translational deficit.

### 6.3 Open challenges of clinical integration

#### 6.3.1 The arrhythmia robustness problem

Since the prediction and control methods of Sections 4, 5 assume quasi-periodicity, all fail under arrhythmia, which is the group most likely to benefit from off-pump procedures. The Tuna et al. (20) estimator was the most systematically evaluated but only for pacemaker-driven rate changes, not the aperiodic distortions of atrial fibrillation (AF), premature ventricular contraction (PVC), or flutter. The Liang et al. (3) Slave-UKF algorithm was developed for aperiodic signals and tested on MIT-BIH PVC (106m) and AF/flutter (203m) records, achieving ECG-signal RMSE of 18.1 and 15.7μV vs. 270.5/167.5μV for AR and 249.4/138.9μV for the Fourier-EKF. However, this was validated on ECG rather than the estimated motion signal, so arrhythmia robustness remains unproven in a closed motion loop; a prerequisite for clinical approval.

#### 6.3.2 The actuation bandwidth constraint

Ginhoux et al. (29) noted that one Aesop joint was too slow to follow the heartbeat at the operating rate, implying general surgical robots are worse. From the motion characterization of Section 2.1.1 (17), following the second harmonic at 200 bpm (6.67 Hz) requires at least 30–40 Hz of bandwidth, roughly ten times the 2–3 Hz Cartesian bandwidth of the commercial Da Vinci, which was not designed for motion compensation. Two research platforms meet the requirement: the Harvard MCI voice-coil actuator (35 Hz) (57) and the LIRMM D2M2 direct-drive robot (35), but neither is clinically certified, so a robot with both CABG dexterity and compensation bandwidth remains an unsolved hardware challenge.

#### 6.3.3 Sensor integration and intraoperative calibration

Clinical tracking faces constraints absent from the lab. Vision requires a clear, calibrated field, conflicting with occlusion, blood, smoke, and specular reflection; Stenmark et al. (44) found 19% of frames unusable due to low operating-room light. 3DUS (7, 57) avoids occlusion but adds 68 ms acquisition delay, and force sensors (34) need no line of sight but give only one degree of freedom. A fused vision–3DUS–force system with an appropriate state estimator may give the most accurate tracking, at the cost of complexity.

#### 6.3.4 The training, learning curve, and adoption barrier

Even without active compensation, adoption is limited by training. Wah (16) reports no accepted curriculum for robotic CABG, and both Balkhy (58) and Baudo and Torregrossa (14) report a 10–50-case learning curve for beating-heart TECAB. Adding a motion-compensation subsystem will raise cost and complexity further, and there is currently no economic model justifying its adoption on existing platforms.

#### 6.3.5 Regulatory and safety architecture

Active compensation introduces a failure mode mechanical stabilizers lack: the robot may move autonomously to an incorrect predicted position, injuring the patient or disrupting an anastomosis, and no current regulatory framework covers this. Shared-control architectures [e.g., Moustris et al. SWAM (55)] are safer than full autonomy but still require evaluation under arrhythmia and prediction failure. Image-guided systems such as those of Unger et al. (59) are further limited by imaging latency (MRI, and likewise vision/ultrasound) in providing real-time feedback.

#### 6.3.6 Force feedback and haptic transparency

As shown in Section 5 (37), the beating heart is a non-passive environment that injects energy into the teleoperation loop, violating the passivity assumption of bilateral haptic design. Commercial systems offer limited haptics: the Da Vinci is visual-only, and the first system with real force feedback (Senhance, Asensus) earned FDA/CE marking only recently, and because the robot drives the motion, the surgeon cannot feel tissue contact or tearing during compensation. Transparent haptic feedback within the active cardiac environment thus remains an open problem (37).

### 6.4 Future directions

#### 6.4.1 Near-term: incremental integration pathways

The most credible near-term route is incremental integration into already-cleared platforms, along three pathways. First, predictive image stabilization as a software layer: the Moustris SWAM framework (9) already runs at 30 Hz on existing hardware with no mechanical change, and Stenmark et al. (44) showed the necessary tracking can be deployed with negligible surgeon inconvenience. Second, hybrid revascularization, the dominant approach to multivessel disease (14), where the MPC catheter of Vrooijink et al. (45) is the most deployable active-compensation method and could integrate with the expanding endovascular platforms reviewed by Duan et al. (60). Third, arrhythmia detection with automatic failsafe: the ECG can detect arrhythmia (3), and its ∼90 ms warning gives time to enter a failsafe before the arrhythmic beat manifests mechanically (3, 6).

#### 6.4.2 Medium-term: sensor fusion and multimodal tracking

The main medium-term effort is sensor fusion, since no single sensor suffices. McClelland et al. (24) found that weighting multiple sensors (ECG, respiratory pressure, image) improves respiratory-motion estimation, and an analogous cardiac model could fuse vision, 3DUS, ECG, and force for the most robust tracking. The NAR network of Cheng and Tavakoli (50) (60% error reduction over EKF at 160 ms) could be extended with online retraining on the patient’s own pattern during the first minutes of surgery, fused with ECG-based initialization and respiratory surrogates.

#### 6.4.3 Longer-term: next-generation robotic platforms and AI integration

Longer-term trends (14, 16) include AI for intraoperative navigation, real-time complication prediction, and automated anastomotic optimization, with the hand-designed EKF/MPC algorithms of Sections 4, 5 potentially learned and adapted to the individual heart intraoperatively. New platforms are emerging beyond Da Vinci: SS Innovations’ SSI Mantra 3 performed the first robotic cardiac surgery in India (14), alongside the Medtronic Hugo RAS, CMR Surgical Versius, and Medicaroid Hinotori, generally with higher bandwidth than the da Vinci Xi; Posselli et al. (61) have also built head-mounted robots compensating patient motion in retinal surgery. Wah (16) further discusses the Internet of Robotic Things, in which the prediction module (Section 4) would draw on preoperative CT geometry, real-time ECG and hemodynamics, and population-level motion databases to initialize a personalized model before incision. More broadly, the move toward surgical autonomy is increasingly driven by robotics, AI, and machine learning (62). Extending this to cardiac compensation would mean learning patient-specific policies online rather than relying on hand-designed EKF/MPC formulations, which raises a further difficulty, since regulatory frameworks for adaptive, continuously-learning algorithms whose autonomy levels fall outside current device clearances do not yet exist (Section 6.3.5).

## 7 Conclusions

### 7.1 Summary of contributions

This review traced beating-heart robotic surgery across four layers. The motion model (Section 2) is best represented as a dual non-stationary Fourier series separating cardiac and respiratory components, with 0.8–1.1 mm residual after mechanical stabilization defining the floor compensation must surpass (31). Prediction (Section 4) spans the Fourier-EKF (0.73–0.81 mm RMSE at 0.18 s (5, 18)), the Master-Slave UKF that uniquely addresses arrhythmia (3), and neural predictors [60% error reduction over EKF at 160 ms (50)]. Control (Section 5) ranges from feedforward servos through GPC disturbance rejection [93% respiratory/80% cardiac variance reduction (29)], cascade MPC-AOB force control [<0.08 N force RMSE (53)], SWAM shared control [41% mean error reduction at 18 bpm (55)], and bilateral teleoperation passivity guarantees (37). In clinical translation (Section 6), the laboratory trajectory from Nakamura’s 2001 prototype through the Harvard group’s in-vivo porcine program (Yuen 2008–2013, Kesner 2011) (2, 6, 7, 57) established feasibility but not human trials with active compensation, while the clinical CABG programs (Balkhy, Lutz, Baudo) executed more than 11,000 MICS-CABG procedures at 95.2% one-year survival (14) using only mechanical stabilization, and live human motion tracking exists only as the ArUco work of Stenmark et al. (44). This gap, in-vivo animal validation achieved, first-in-human not yet initiated, is the field’s defining translational deficit.

### 7.2 Synthesis: the system-level view

A cross-cutting theme is that performance is limited by the weakest link: an accurate predictor is wasted on a high-latency sensor, the best force controller cannot act beyond the robot’s joint bandwidth, and the best-validated motion model fails during arrhythmia without failsafe logic. The positive corollary is that improving any one component lifts the whole system. For example, lower sensing latency improves both prediction and force regulation, and better prediction can simplify the control architecture. Yuen et al. (57) illustrate this: their prediction error (1.7 mm) and control error (1.8 mm) were nearly equal, so further gains require improving either component, underscoring the need for system-level co-design.

### 7.3 Open challenges and future directions

The evidence base has limitations. Most pre-clinical validation used porcine models under controlled ventilation; dataset durations ranged from 15 s (18) to about 1000 s for neural-network training (50), but none spans a full multi-hour procedure, and performance under arrhythmia is largely unstudied (3, 6). The review itself is limited by single-reviewer screening (a potential selection bias), an English-language restriction (possible omission of non-English work), and approximate rather than exact record counts. Section 6 identified six open challenges being arrhythmia robustness, actuation bandwidth, sensor fusion, training and adoption economics, regulatory certification, and haptic transparency, and four research directions: personalized intraoperative motion models, multimodal Bayesian sensor fusion, integration with catheter-based robotics, and shared control with formal safety certification. These challenges differ in kind: engineering problems (arrhythmia robustness, bandwidth) are solvable with resources; haptic transparency in the active cardiac environment needs new mathematical frameworks; and regulatory certification is primarily institutional and procedural. Recognizing these differences helps prioritize how the field addresses each.

The proposed roadmap has limitations of its own: its recommendations are synthesized from a porcine-dominated literature rather than validated experimentally, and the benchmark assumes multi-institutional data sharing that faces real ethical and proprietary barriers. Future work should prioritize prospective validation of an integrated pipeline on human data, establishment of the benchmark and its governance, and formal safety-case development for shared-control compensation.

### 7.4 Concluding remarks

Robotic-assisted beating heart surgery stands at a well-characterized technical frontier: the prediction algorithms, control architectures, and sensing modalities for sub-millimeter tracking have each been demonstrated pre-clinically, and commercial robots have proven minimally invasive cardiac surgery viable. A central insight is that the principal remaining gap is one of integration rather than of any single missing algorithm. This does not deny that technical challenges remain. For example, actuation bandwidth and arrhythmia robustness (Section 6.3) are genuine unsolved problems, but these are now well-defined engineering targets, and the dominant barrier is the absence of an integrated, validated system rather than the immaturity of any component.

From this synthesis, four practical recommendations follow for groups seeking first-in-human trials. First, prioritize systems integration over isolated algorithmic refinement: couple an already-validated predictor with an ECG-triggered arrhythmia failsafe and a real-time sensing front end. Second, adopt shared or canonical-stabilization control (Section 5.4) for the initial safety case, deferring higher-autonomy closed-loop compensation until the regulatory questions of Section 6.3.5 are resolved. Third, pursue predictive image stabilization as a software layer on an already-cleared platform (Section 6.4.1) as the lowest-regulatory-risk entry point. Fourth, establish a shared multi-institutional benchmark dataset, since the absence of such data, not algorithmic capability, is now the binding constraint on validation. It should provide synchronized recordings of the key modalities (stereo-endoscopic video, 3D ultrasound, ECG, respiratory surrogates, and contact force) with high-accuracy ground-truth 3D position, spanning both sinus rhythm and annotated arrhythmia across species and human data. Standard metrics should include prediction RMSE vs. horizon, arrhythmia-detection latency, force-regulation error, computational latency, and cross-patient generalization. Figure 9 summarizes the four barriers separating pre-clinical success from clinical deployment. Multiple new robotic platforms now offer the joint bandwidth needed for active compensation; whether the robotics, surgical, and regulatory communities converge on a shared validation framework will determine whether this capability reaches patients, the ultimate objective being to let a surgeon interact with the beating heart as though it were stationary, which is the subject of our ongoing work.

**Figure 9:**
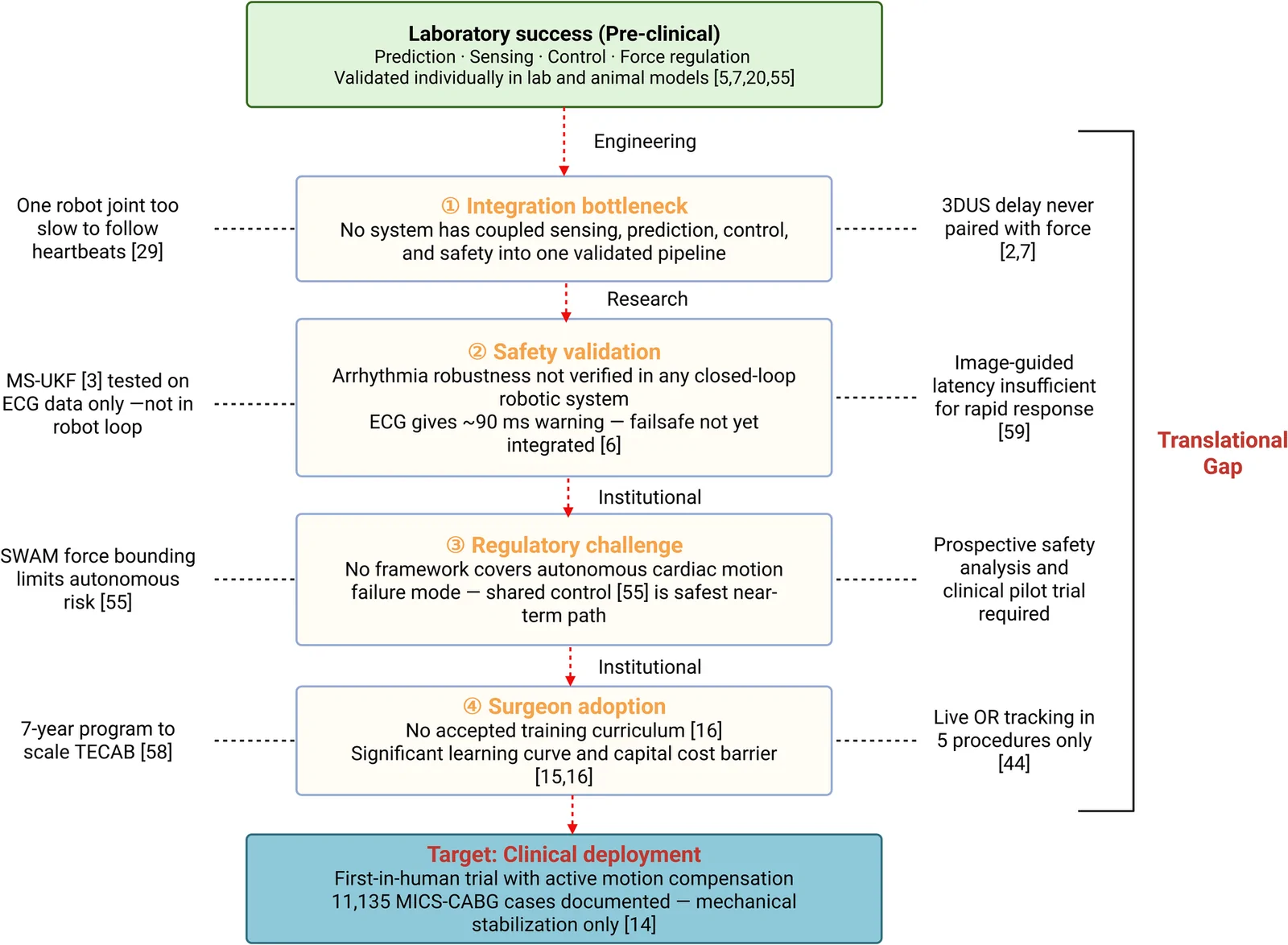
The translational gap in robotic beating heart surgery.

## Data Availability

The original contributions presented in the study are included in the article/Supplementary Material, further inquiries can be directed to the corresponding author.
